# Configuring a Liquid State High‐Entropy Metal Alloy Electrocatalyst

**DOI:** 10.1002/smll.202504087

**Published:** 2025-06-17

**Authors:** Sahar Nazari, Ali Najmi, Priyank Kumar, Ali Zavabeti, Francois‐Marie Allioux, Anirudh Raju Natarajan, Dorna Esrafilzadeh, Ali R. Jalili

**Affiliations:** ^1^ School of Chemical Engineering University of New South Wales (UNSW) Sydney NSW 2033 Australia; ^2^ School of Civil and Environmental Engineering University of New South Wales (UNSW) Sydney NSW 2033 Australia; ^3^ Department of Chemical Engineering RMIT University Melbourne VIC 3001 Australia; ^4^ School of Chemical and Biomolecular Engineering The University of Sydney Sydney NSW 2033 Australia; ^5^ Laboratory of Materials Design and Simulation Institute of Materials École Polytechnique Fédérale de Lausanne Lausanne 1015 Switzerland; ^6^ Graduate School of Biomedical Engineering University of New South Wales (UNSW) Sydney NSW 2035 Australia; ^7^ School of Chemistry University of New South Wales (UNSW) Sydney NSW 2052 Australia

**Keywords:** design of experiment, DFT, electrocatalysis, green ammonia, high‐entropy liquid metal alloy

## Abstract

A high‐entropy liquid metal alloy (Ga–Fe–Zn–Sn–Bi–Ni) is developed to address the multi‐step complexity of green ammonia electrosynthesis from nitrate. Guided by molecular dynamics, design of experiments, and density functional theory, this alloy exploits high configurational entropy to form diverse, atomically dispersed active sites. The liquid state eliminates endothermic barriers by enabling nitrogen intermediates to move freely to the most energetically favorable sites. Crucially, a hydrogen shuttling mechanism is uncovered where Fe acts as a proton hub while Sn, Ni, and Zn store and transfer hydrogen to Fe, enhancing reaction kinetics and preventing catalyst saturation. This synergy boosts ammonia production rates up to sevenfold while maintaining high Faradaic efficiency (FE). By integrating entropy‐driven design, dynamic site reconfiguration, and hydrogen management, this work establishes a robust foundation for efficient, scalable ammonia electrosynthesis in pursuit of NetZero targets.

## Graphical abstract

A liquid‐state high‐entropy metal alloy is developed to overcome key kinetic bottlenecks in ammonia electrosynthesis from nitrate. Atomically dispersed active sites and a dynamic hydrogen‐shuttling mechanism enable barrier‐free deoxygenation and hydrogenation. Guided by molecular dynamics, design of experiments, and density functional theory, the alloy achieves up to 316 nmol s⁻¹ cm⁻^2^ ammonia production at near‐quantitative efficiency. This study establishes liquid high‐entropy electrocatalysts as a robust and scalable platform for green ammonia generation.

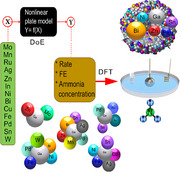

## 1 Introduction

Global dependence on fossil fuels has produced substantial CO₂ emissions, driving the urgent need for decarbonization through renewable energy sources.^[^ ^1^ ^]^ Ensuring effective storage of renewable energy is vital, with hydrogen and green fuels like ammonia playing central roles. As both a hydrogen carrier and a fertilizer, ammonia enhances energy security while curbing carbon emissions.^[^ ^2^, ^3^ ^]^ Its existing infrastructure can store ≈1.2 PWh annually, enabling large‐scale, long‐term storage.^[^ ^4^ ^]^ Established liquefaction, storage, and transportation methods further demonstrate its scalability.^[^ ^5^ ^]^ To decarbonize ammonia production, we must replace conventional, emission‐intensive methods with decentralized, renewably powered systems.^[^ ^6^ ^]^ While electrochemical nitrogen reduction is complicated by nitrogen's inertness, the electrochemical reduction of nitrogen oxides or (NO_x_RR) presents a more accessible path, circumventing high energy barriers. Crucially, nitrates can be generated via non‐thermal plasma, providing high productivity and supporting the scalability of this approach.^[^ ^7^, ^8^ ^]^

Despite its potential, NO_x_RR is inherently complex, involving multiple reaction steps and electron transfers.^[^ ^9^ ^]^ Density functional theory (DFT) studies associate its activity with the adsorption energies of intermediates like ^*^NO, ^*^NO₂, and ^*^NO₃⁻, revealing multiple pathways and rate‐limiting steps.^[^ ^10^ ^]^ Although tuning catalyst properties to optimize free energies and strengthen intermediate interactions is critical, it remains challenging.^[^ ^11^ ^]^ For nitrate (NO₃⁻) reduction specifically, supplying eight electrons and ten protons to remove three oxygen atoms as water before adding three protons makes hydrogen availability a key factor.^[^ ^7^ ^]^ Moreover, since the proton is consumed ten times faster than nitrate, ensuring efficient hydrogen delivery emerges as a significant bottleneck, often overshadowed by efforts to enhance nitrogen adsorption and reactivity. Thus, effective catalysts must balance NO_x_⁻ reduction with hydrogen supply, minimize H₂ formation, and prevent catalyst saturation. Metal oxides support the hydrogen evolution reaction by favoring N‐based reactions, which also slows hydrogen delivery to ^*^N intermediates, limiting their reactivity.^[^ ^12^, ^13^ ^]^ Addressing these challenges calls for improved hydrogen management, continuous oxygen removal, and catalysts that bind and reduce nitrogen effectively and maintain a steady hydrogen supply.^[^ ^14^ ^]^

Enhancing such cascade electrocatalytic processes depends on developing versatile catalysts with multiple bonding energies, each acting as an independent catalytic unit.^[^ ^15^ ^]^ High‐entropy alloys (HEAs), which integrate five or more elements into a single stable phase, offer diverse design possibilities for optimizing catalytic activities by creating active sites with optimal binding energies for multiple intermediates.^[^ ^15^, ^16^, ^17^, ^18^ ^]^ However, traditional HEAs face challenges due to numerous combinations, segregation, and synthesis complexities, making systematic element addition or removal difficult without altering surface properties like surface area and nanostructure.^[^ ^19^, ^20^, ^21^, ^22^ ^]^ Theoretical studies, including DFT and machine learning, aid in predicting stable structures and reaction pathways for HEAs.^[^ ^23^, ^24^ ^]^ but often fail to capture real‐world complexities such as kinetics and environmental influences.^[^ ^25^, ^26^ ^]^ This necessitates strategies to navigate the compositional space and develop synthesis protocols that yield catalysts with consistent properties.

Liquid metals like gallium enable the incorporation of multiple transition metals into a single, stable, atomically dispersed phase, facilitating HEAs.^[^ ^27^ ^]^ Recent advances in liquid‐state high‐entropy liquid metal alloy (HELMA) have confirmed the unique catalytic advantages that emerge when multicomponent metals are kept in the fluid state. Pioneering Ga‐based systems now span reactions from CO₂ electro‐reduction to oxygen evolution,^[^ ^27^ ^]^ where the liquid matrix entropically stabilizes single‐atom active centers, suppresses phase segregation, and enables self‐healing against poisoning. Operando spectroscopy and TEM have further revealed sub‐nanosecond atomic reshuffling at the interface, rationalizing the exceptionally low activation barriers predicted by theory. Collectively, these studies highlight HELMA as a distinct materials class whose dynamic surfaces, high charge mobility, and tailorable local bonding environments can unlock reaction pathways inaccessible to conventional solid catalysts.

Recent studies have demonstrated a high activity of gallium‐based liquid metal for NO_x_RR to ammonia.^[^ ^28^ ^]^ However, these studies typically identify solidified intermetallic sites as primary catalysts, making using liquid‐state electrocatalysts as HEAs unprecedented. In the liquid state, metal alloy catalysts offer enhanced charge delocalization and atomic diffusion, providing superior properties compared to solid alloys. Configuring a HELMA electrocatalyst is crucial, as the liquid state maintains a consistent alloy structure, allowing the addition or removal of elements without significantly altering surface properties, unlike solid catalysts that form different shapes or surface areas.^[^ ^29^ ^]^ This approach isolates entropy and catalytic effects, enabling flexible alloy design and dynamic interactions with intermediates, while preventing trapped particles and ensuring a homogeneous catalyst surface.

Here, we present an integrated framework for designing a high‐performance HELMA electrocatalyst for ammonia synthesis. Using molecular dynamics (MD) simulations, the ratios of added metals to gallium required to maintain liquid properties near room temperature are determined. Building on these insights, we then employed a design of experiments (DoE) approach,^[^ ^30^ ^]^ systematically adding and removing 13 transition metals (Mo, Mn, Ru, Ag, Zn, In, Ni, Bi, Cu, Fe, Pd, Sn, and W) to refine the alloy's composition with minimal experimentation. By iterating between MD simulations, DoE‐based optimization, and experimental validation within a synergistic feedback loop, we identified an optimal five‐element HELMA catalyst. Comprehensive DFT modeling clarified each element's contribution to the reaction cascade, enabling the fine‐tuning of hydrogen adsorption and nitrogen reduction steps. In the liquid state, multiple bonding environments emerged, reducing energy barriers and creating specialized active sites for every reaction step, while efficiently shuttling hydrogen.

## 2 Results and Discussion

### 2.1 Synthesis and Optimization of Liquid Metal Alloys

**Figure 1:**
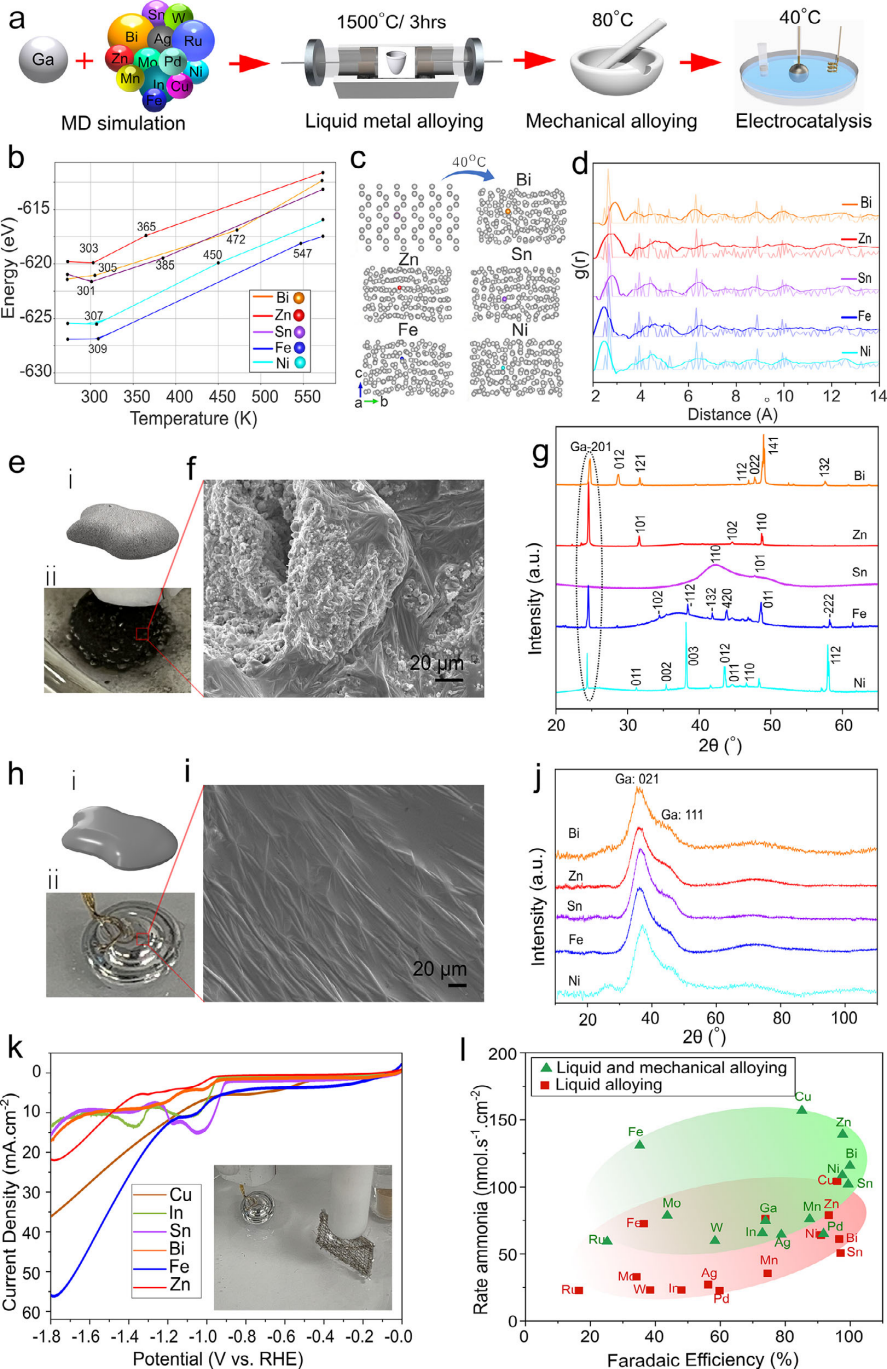
A liquid metal alloying process enabling high‐entropy atomic‐level dispersion of elements. a) Schematic of the alloying process: MD simulations predict alloy compositions, followed by adding a selected metal to gallium. The mixture is melted at 1500 °C for 3 h and then cooled to 80 °C. Mechanical mixing with a mortar and pestle redistributes aggregates, repeated incrementally every 10 °C down to 40 °C. b) Segmented regression analysis for binary alloys containing 0.5 wt.% metal, derived from MD simulations over a broad temperature range. c) Structural changes in various binary alloy systems modeled by MD simulations as temperature increases, indicating phase transformations to a liquid state occurring ≈300 K. d) Radial distribution functions g(r) of binary alloys (0.5wt.%) at 40 °C (dark‐colored) and the inherent structures before transition to liquid (light‐colored). e‐i) Schematic, e‐ii) snapshot of the liquid alloy without mechanical mixing in the electro‐cell, and f) a typical SEM image of the surface of these aggregated alloys, with XRD spectra in g. h‐i) Schematic, i‐ii) snapshot of liquid alloy with mechanical mixing in the electro‐cell, j) a typical SEM image of the surface, and XRD spectra of the corresponding alloys, showing atomic dispersion. k) LSV of selected binary alloys in nitrate medium; inset shows electrocatalysis setup with liquid alloy. l) Production rate and FE of alloys when used for ammonia synthesis, color‐coded: red for liquid alloying only, green for liquid alloying, plus mechanical mixing.

Alloys were synthesized following the procedure outlined in **Figure** **1**a. Phase diagrams (Figure S1, Supporting Information) indicate the range of compositions that can form liquid alloys near room temperature. We performed MD simulations to identify the maximum concentration of secondary metals that maintains the liquid state within this range. The simulations tested secondary metal concentrations of 0.25, 0.5, 0.75, 1, and 1.5 wt.% relative to gallium (Figures S3–S6, Supporting Information). The results revealed that 0.5 wt.% is the highest concentration at which all binary alloys remained liquid near room temperature. Figure 1b,c presents the MD simulation results and the segmented regression analysis for binary alloys containing 0.5 wt.%. The energy–temperature relationship derived from these simulations reveals a significant structural transition near room temperature, indicative of a solid‐to‐liquid phase change. Figure 1d shows the radial distribution functions (g(r)) for these alloys. The lighter‐colored curves represent the initial state, where the material remains solid. In contrast, the darker‐colored curves, obtained after heating to 40 °C, exhibit no sharp peaks and a more flattened profile. This change demonstrates the liquefaction transition.

However, under experimental conditions, skin formation was observed during cooling, leading to segregation and the development of a metal oxide layer on the alloy surface, which resulted in the appearance of black skin during electrochemical tests (Figure 1e). Scanning electron microscopy (SEM) of the cooled alloy surfaces (Figure 1f) revealed the presence of surface particulates. X‐ray diffraction (XRD) analysis of these aggregates (Figure 1g) reflects peaks from Ga (‐201) as well as the formation of distinct metallic and intermetallic phases between Ga and each second metal, with peak positions and intensities confirming that the aggregates comprise metal‐rich crystals of secondary metals on the surface. To address this issue, we improved our preparation method by manually milling the alloys at 80 °C, followed by stepwise milling down to 40 °C inside a glove box using a mortar and pestle, rather than directly cooling the melt to near room temperature. This mechanical milling process played two critical roles: it effectively removed surface oxides and ensured thorough homogenization of the constituent metals, thereby mitigating aggregation and uncontrolled solidification. Figure 1h shows a snapshot of the resulting liquid alloy employed as a cathode in our electrocatalysis tests. Figure 1i presents an SEM micrograph of a typical liquid alloy surface, revealing no visible aggregates or black skin. The corresponding XRD spectra (Figure 1j) confirm the alloy's uniform distribution, as indicated by the absence of metallic peaks. This absence could reflect complete atomic‐level dispersion, the removal of aggregates through milling, or the migration of any residual clusters beneath the XRD penetration depth. Since electrocatalysis involves only the exposed outer surface, any aggregates that may remain beneath the surface and within the bulk have minimal to no impact on the alloy's inherent catalytic performance.

Figure 1k illustrates the linear sweep voltammetry (LSV) of selected binary alloys in nitrate medium, showing a consistent increase in current density as the potential decreases to −1.8 V versus RHE, followed by a broad redox peak area starting from −0.8 V versus RHE. This is indicative of the electrochemical conversion of NO₃⁻ to NH₄⁺, which proceeds via two pathways.^[^ ^31^ ^]^ The first pathway involves the direct conversion of NO₃⁻ to ammonia, exchanging eight electrons at overpotentials lower than the hydrogen evolution reaction (Equations 1 and 8), while the second pathway involves the reduction of NO₃⁻ to NO₂⁻, which is further reduced to NH₄⁺ (Equations 9 and 10). These peaks align with previous studies and suggest a two‐step reduction mechanism, with the NO₂⁻ intermediate pathway becoming more pronounced at overpotentials higher than −0.8 V versus RHE. Both pathways can occur simultaneously, depending on catalyst and electrolyte conditions, and ongoing work is focused on optimizing these processes for better catalytic efficiency.

Figure 1l illustrates the ammonia production rate versus FE for all binary alloys tested, categorized into atomically dispersed (green shadow) and aggregated states (red shadow), to highlight the impact of the alloying method on catalytic performance. The findings show that alloys created through mechanical mixing and melting performed noticeably better than those produced by melting alone. Preventing aggregation nearly doubled the ammonia production rate for each alloyed metal, with a moderate impact on FE.

### 2.2 Design of Experiments for Efficient Catalyst Optimization

**Figure 2:**
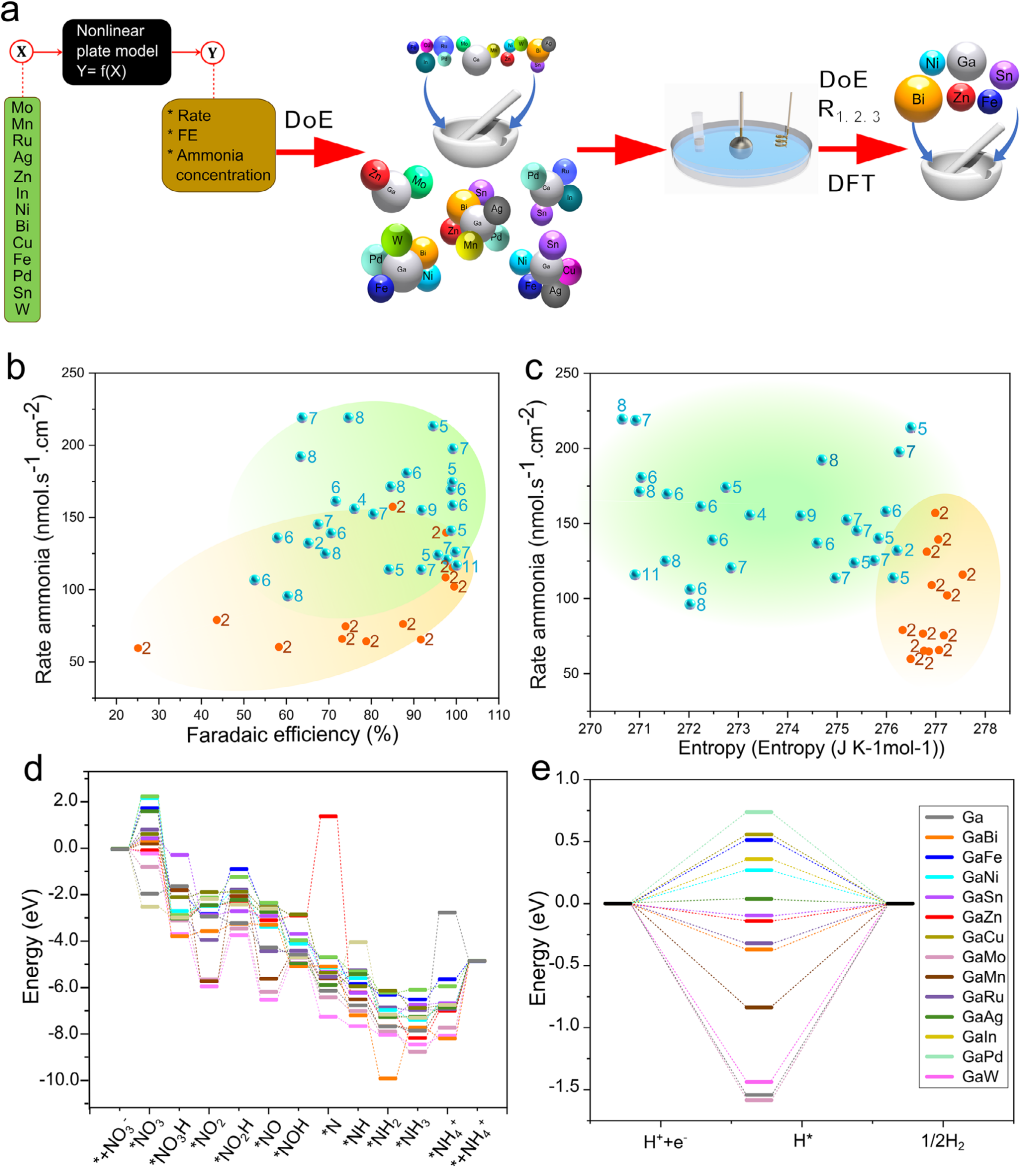
DoE methodology is integrated with a theory‐experimental feedback loop. a) Schematic illustration of the DoE procedure for selecting and producing multi‐component alloys. b,c) Ammonia production rate versus FE and entropy, respectively, color‐coded: brown for binary alloys and green for multi‐component alloys. Numbers indicate the number of elements in each composition, including gallium. d) DFT‐based free energy diagram illustrating the NO_x_RR mechanism on the 13 alloys. e) Corresponding hydrogen evolution reaction free energy diagram for the same alloys.

Adopting DoE in this study provides a highly efficient framework for navigating the complex, multidimensional landscape of electrocatalyst design. **Figure** **2**a summarizes the systematic application of DoE principles to optimize the catalyst mixture, emphasizing how the methodology strategically avoids the pitfalls associated with conventional exhaustive testing. Instead of undertaking an impractical complete factorial design (2¹^3^  =  8192 experiments) with 13 elements, we leveraged DoE to select 28 carefully chosen alloy compositions (Table S1, Supporting Information). These compositions were sufficient to identify meaningful trends, interactions, and non‐linear relationships, thereby distilling critical insights from a significantly reduced experimental effort. By defining ammonia concentration, production rate, and FE as key performance targets and carefully controlling all other experimental parameters, the DoE approach minimized confounding factors. It maintained a strong focus on the variables of interest. Such a structured method ensured that the chosen alloys provided robust statistical coverage of the design space, capturing both main effects and higher‐order interactions that would remain obscured under one‐factor‐at‐a‐time or purely trial‐and‐error strategies.

Figure 2b compares the ammonia production rate versus FE for all combinations, distinguishing between binary alloys (light brown) and multi‐element liquid alloys (green) (see also Table S2, Supporting Information). The results show that multi‐element liquid alloys exhibit superior ammonia production rates, with three compositions achieving over 200 nmol cm⁻^2^ s⁻¹, significantly higher than most binary alloys, which range between 50 and 100 nmol cm⁻^2^ s⁻¹. Figure 2c plots the ammonia production rate against calculated entropy, revealing that binary alloys often exhibit higher entropy than multi‐element liquid alloys. The hard sphere model stems from optimal atomic size ratios that enhance packing efficiency and maximize packing entropy, coupled with minimal liquid phase separation (LPS) and chemical short‐range ordering (SRO) in binary systems.^[^ ^32^ ^]^ Binary alloys benefit from similar atomic sizes, producing high packing fractions.

In contrast, multi‐component systems with disparate sizes suffer from packing inefficiencies and reduced entropy due to LPS or SRO.^[^ ^33^, ^34^ ^]^ Although configurational entropy increases with more components,^[^ ^35^ ^]^ non‐additive effects and the model's neglect of enthalpic interactions can overestimate binary entropy and underestimate multi‐component entropy, particularly when complex interactions disrupt mixing.^[^ ^36^, ^37^ ^]^ Ammonia production in NO_x_RR does not solely depend on high entropy, as some multi‐element liquid alloys with lower entropy outperform their higher‐entropy binary counterparts, highlighting the critical interplay of entropic and synergistic effects. High configurational entropy stabilizes the liquid phase, maintaining a homogeneous, single‐phase alloy with uniformly distributed active sites. In contrast, the liquid state amplifies this by enabling dynamic reconfiguration of catalytic sites. Synergistic interactions among elements, such as hydrogen shuttling, hydrogen management, and stabilization of nitrogen intermediates, drive superior performance. In atomically dispersed liquid alloys, these specific elemental interactions and active site configurations often outweigh the entropy effect, underscoring that synergy primarily fuels catalytic efficiency, while entropy ensures stability, forming a robust platform for sustainable ammonia synthesis.

### 2.3 Synergy of Computational Modeling and Experimental Optimization

We performed DFT modeling on each of the 13 elements individually alloyed with gallium to understand why binary liquid alloys, despite their higher entropy, do not achieve a better performance. The adsorption energies of nitrate and its intermediates on each metal within the liquid matrix were computed. Figure 2d presents the free energy diagrams for the NO_x_RR mechanism on the 13 binary liquid alloys and pure gallium. The NO_x_RR process involves 13 steps (see Equations (8), (9), (10), (11), (12), (13), (14), (15), (16), (17), (18), (19), (20)) of deoxygenation and hydrogenation, summarized by the reaction:

(1) $${\mathrm{NO}}_{3}^{-}+10H^{+}+8e^{-}\to {\mathrm{NH}}_{4}^{+}+3H_{2}O$$

The initial nitrate adsorption step requires that hydrogen adsorption does not dominate the surface, as excessive hydrogen adsorption can lead to surface saturation, inhibiting nitrate ion adsorption (Figure 2e). Subsequent reduction steps, such as forming NO₃H^*^ and NO₂^*^, are energetically endothermic for most metals, representing significant rate‐determining steps (Table S3, Supporting Information). These barriers hinder high‐rate ammonia synthesis, explaining why binary alloys, even with high entropy, did not outperform multi‐element alloys with lower entropy. Consequently, we focused on identifying optimal HELMAs with increased entropy and well‐mixed, fine‐tuned combinations of elements.

**Figure 3:**
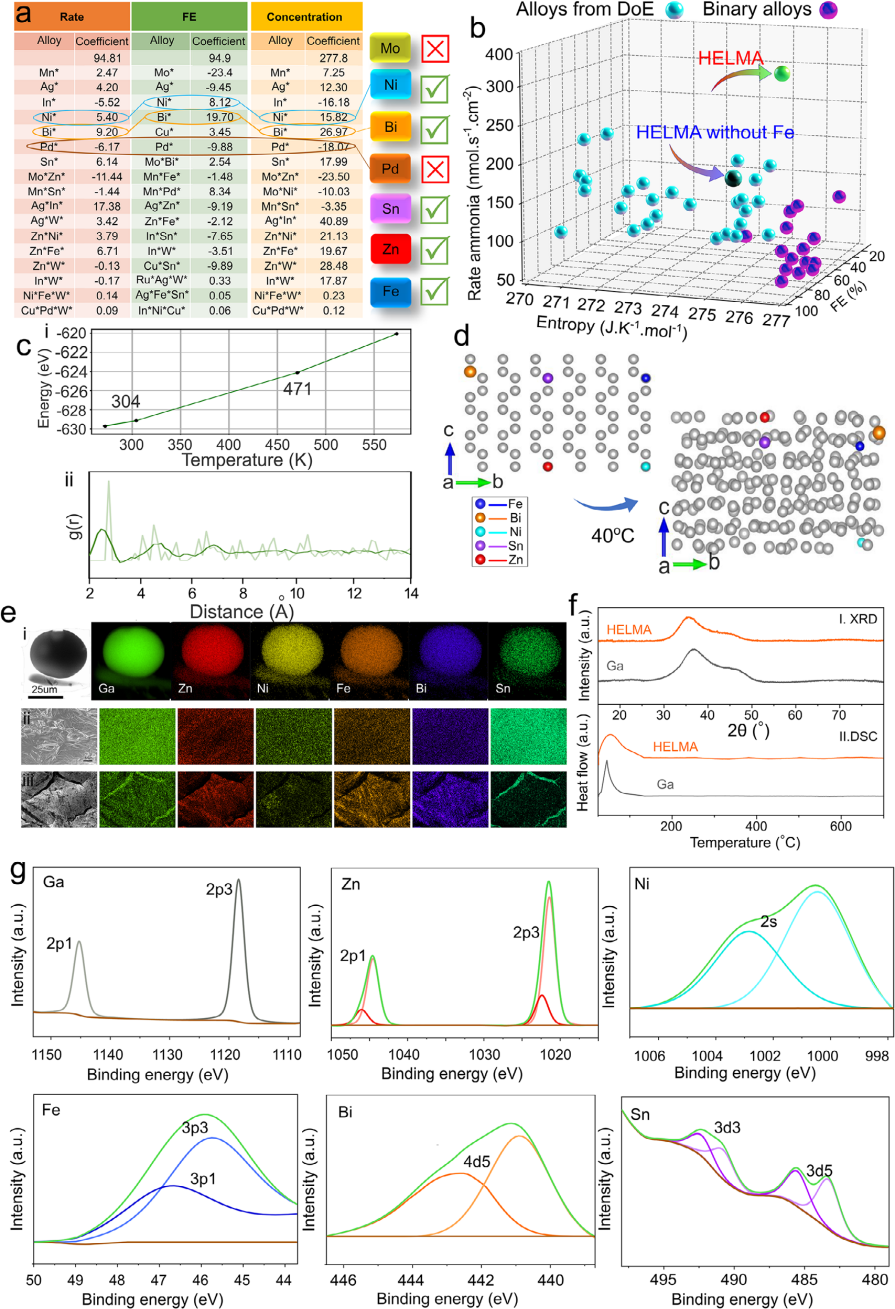
The DoE‐driven selection mechanism to refine multi‐component alloys to optimize a high‐entropy catalyst for enhanced ammonia synthesis performance. a) Snapshot of the table highlighting elements with positive responses to be included and negative responses to be excluded in the final alloy. As highlighted, elements like Pd and Mo show negative responses, while Ni, Bi, and Fe exhibit positive responses. b) Ammonia production rate versus entropy and FE for all initial alloys suggested by DoE (blue) and binary alloys (purple); the final optimized HELMA, identified through DoE analysis, is highlighted in green. c‐i) Segmented regression analysis for HELMA, derived from MD simulations over a broad temperature range, and c‐ii, radial distribution functions g(r) of HELMA at 40 °C (dark‐colored) and the inherent structures before transition to liquid (light‐colored). d) The structural changes in HELMA systems as temperature increases to 40 °C. e‐i) TEM micrograph and EDS analysis of a small liquid droplet of the final HELMA catalyst. e‐ii, iii) SEM images of the catalyst surface before and after ammonia synthesis, respectively, with EDS analysis. f) XRD i) EDS ii) and g) XPS analysis of the electrocatalyst shows all elements constituting HELMA on the surface.

Using the experimentally obtained NO_x_RR performance metrics from 28 alloy combinations, we calculated three response factors: ammonia production rate (R_1_), Faradaic efficiency (R_2_), and ammonia concentration (R_3_), employing multiple non‐linear regression as shown in Equation (2).

(2) $$R=\beta_{0}+\sum_{i=1}^{13}\beta_{i}.Element_{i}+\sum_{i=1}^{13}\sum_{j=i+1}^{13}\beta_{ij}.\left(Element_{i}\times Element_{j}\right)+\cdots +\varepsilon$$

where β_0_ is the constant, β_i_ are coefficients for the main effects, and β_ij_ are coefficients for the interaction effects between each pair of elements. These response factors (R_1_, R_2_, and R_3_) were analyzed to determine the inclusion of different elements based on their effectiveness and chemical and physical interactions. **Figure** **3**a illustrates the selection mechanism using R_1‐3_ responses and calculated figures for multi‐component alloys.

Analysis of responses from 28 combinations identified a final HELMA, comprising Fe, Bi, Ni, Sn, and Zn, as the most effective alloy for enhancing ammonia synthesis, significantly outperforming the individual performance of its components and all other multi‐component combinations. Figure 3b is a 3D scatter plot that compares the entropy, FE, and rate of ammonia production for alloys sourced from the DoE (blue), binary alloys (purple), and HELMA with (green) and without (black) Fe. The plot reveals binary alloys cluster at lower entropy values and exhibit a wide range of ammonia production rates. This indicates that their performance is highly variable and generally limited by lower configurational entropy. Alloys from DoE show a broader distribution of entropy values and ammonia production rates, suggesting improved performance due to increased compositional complexity. HELMA (green sphere) achieves the highest ammonia production rate (316 nmol·s⁻¹·cm⁻^2^) at an entropy of ≈276 J·K⁻¹·mol⁻¹ and an FE of roughly 100%, underscoring the synergistic effect of high entropy and Fe in enhancing catalytic activity. In contrast, HELMA without Fe (black sphere) exhibits a significantly lower ammonia production rate (147 nmol·s⁻¹·cm⁻^2^) at an entropy of ≈275 J·K⁻¹·mol⁻¹ and an FE of 81.2% despite a similar entropy value, with its position closer to the DoE alloys. This comparison demonstrates Fe's critical role in boosting the ammonia production rate in HELMA, likely due to Fe's ability to facilitate active site formation for the H transfer pathway and enhance electronic interactions within the high‐entropy matrix, which are absent in the Fe‐free control. Notably, the optimized catalyst does not possess the highest entropy, indicating that catalytic activity and synergistic effects among constituent metals, while in the liquid state, are more crucial than entropy alone. Figure 3c‐i displays the segmented regression analysis for HELMA based on MD simulations. The energy–temperature relationship derived from the MD simulations confirms that the alloy remains in a liquid state above 304 K. Figure 3c‐ii shows that the g(r) function demonstrates the liquefaction transition at 40 °C. Figure 3d illustrates the structural changes within this temperature range (see also Figure S5, Supporting Information), highlighting the atomic dispersion of elements and demonstrating the transition to a liquid phase without any segregation or aggregation.

Figure 3e‐i presents a TEM micrograph with corresponding EDS analysis of the final HELMA catalyst in its liquid droplet form, confirming that all elements are atomically dispersed within the gallium matrix without forming segregated metallic particles. Pre‐ and post‐reaction SEM images (Figure 3e‐ii,iii), along with EDS, further demonstrate the absence of any significant clustering. Similarly, the XRD pattern (Figure 3f‐i) lacks metallic peaks, indicating a uniform dispersion and no particle formation. DSC analysis (Figure 3f‐ii) reveals a broad melting peak just above room temperature, experimentally validating the MD simulation results and confirming the formation of a single‐phase amorphous alloy with no evidence of aggregation or particle migration into the core. XPS analysis (Figure 3g) confirms the presence of all five elements on the surface.

### 2.4 Dynamic Reaction Pathways Enabled by Liquid‐State Mobility in HELMA

**Figure 4:**
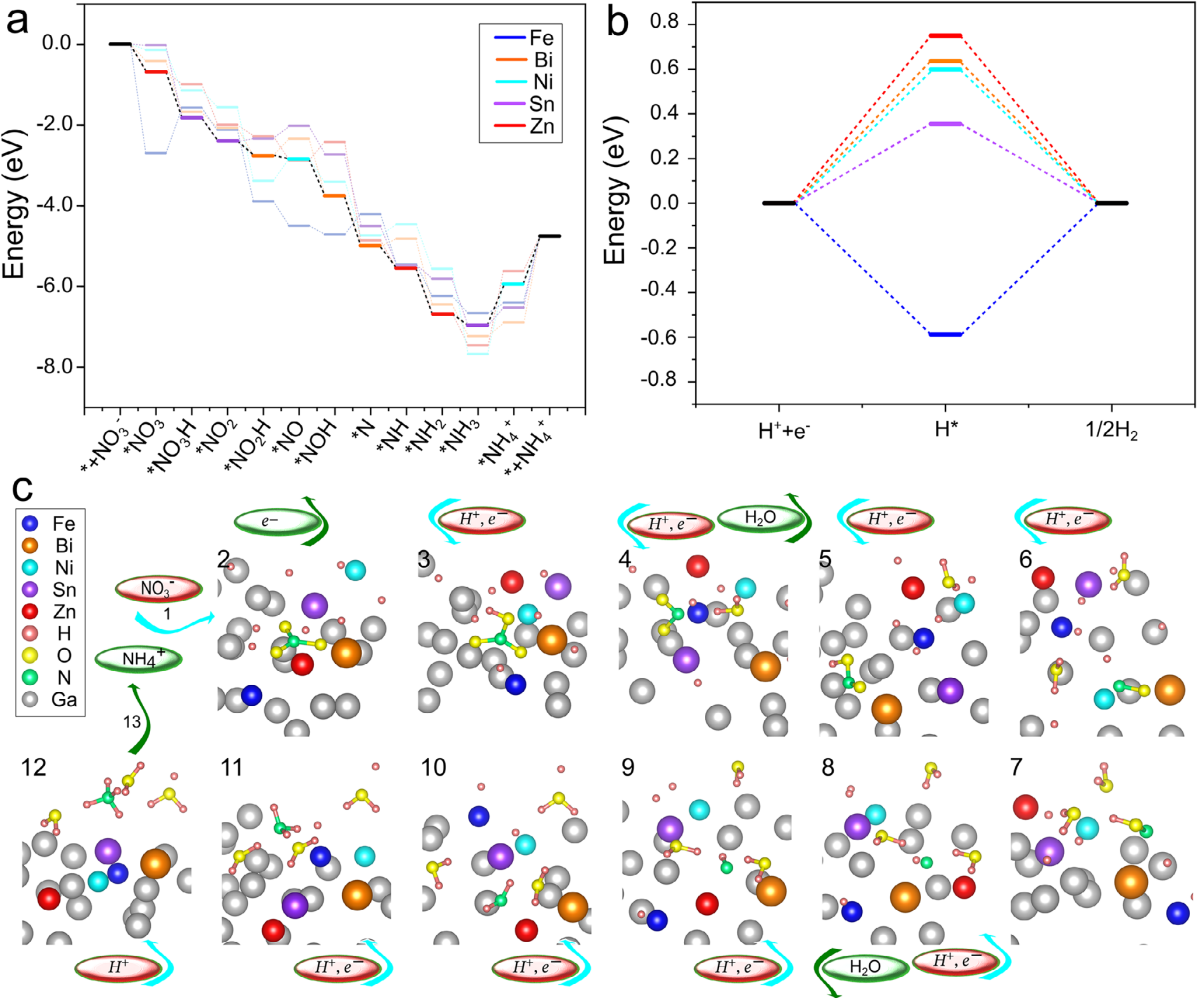
DFT modeling reveals the role of high‐entropy and dynamic environments in enabling energy barrier‐free ammonia electrosynthesis while hydrogen shuttling. a) DFT calculation results for HELMA, each reaction pathway is color‐coded to indicate the central and active elements at each step. b) Corresponding free energy diagram for hydrogen absorption. c) Schematic of the calculated reaction pathway, modeled as slabs containing 32 atoms, including Ga, nitrate, one atom from each of the five metals (Fe, Sn, Zn, Ni, Bi), and ten hydrogen atoms.

**Figure** **4**a,b illustrates the DFT‐calculated energy profiles, reaction pathways, and the corresponding free energy diagram for hydrogen adsorption in HELMA, respectively. Figure 4c presents the reaction pathway identified through DFT modeling, highlighting how the liquid state and elemental mobility within HELMA facilitate catalytic interactions at optimal sites during each stage (see also Movie S1, Supporting Information). Performing DFT calculations for multi‐element catalysts is highly challenging, and the liquid state and mobility of the catalyst constituents further increase this complexity. To address this, we developed a novel procedure to understand and predict the most probable configurations in which intermediates interact with the catalyst and identify which elements are involved. The Supporting Information and Table S4 (Supporting Information) provide a comprehensive discussion of this procedure.

In the initial step, nitrate (^*^NO₃) adsorbs onto the Zn surface, reducing the free energy by −0.68 eV. During the first hydrogenation, the ^*^NO₃H intermediate migrates from Zn to Sn, further lowering the free energy to −1.8 eV. The fourth step involves eliminating the first oxygen atom as a water molecule forms, leaving ^*^NO₂ adsorbed near Sn and decreasing the free energy to −2.4 eV. Without the migration of the nitrogen atom from Zn to Sn during the first deoxygenation, the free energy would have been 0.4 eV higher. Additionally, our DFT modeling suggests that if ^*^NO₂ were to interact with Ni at this stage, the reaction could terminate by releasing nitrite ions instead of proceeding via an adsorbed ^*^NO₂, potentially reducing the Faradaic efficiency; a low‐efficiency reaction pathway that we have previously experimentally reported^[^ ^7^, ^8^ ^]^ and in this work was circumvented. A liquid multi‐element synergistic system allows us to configure the reaction pathway effectively. This was facilitated by the liquid state and high elemental mobility of HELMA, ensuring optimal catalytic performance by allowing active sites to adapt dynamically throughout the reaction process.

Deoxygenation proceeds through Equations (11) and (12) by eliminating a second oxygen atom. First, the ^*^NO₂H bond forms on Bi, lowering the free energy to −2.76 eV, then forming ^*^NO on Ni, further reducing the free energy to −2.85 eV. In steps (13) and (14), the final oxygen atom is removed as the nitrogen core interacts with Bi, resulting in ^*^N bonded to Bi on the surface and decreasing the free energy to −4.74 eV. During Equations (15) and (16), Zn catalyzes the addition of two hydrogen atoms to the nitrogen core, reducing the free energy to −5.56  and −6.68 eV, respectively. In Equation (17), Sn catalyzes the formation of ammonia (^*^NH₃) with a free energy of −6.98 eV. Under the acidic pH conditions of our experiments, the reaction proceeds to form ^*^NH₄⁺, then is released (Equations (18) and (19), respectively). This dynamic environment enables the system to relax and reach equilibrium at each step, eliminating energy barriers by allowing elements and intermediates to move freely across the liquid catalyst. This flexibility, uniquely achievable in a liquid‐state catalyst with numerous small, single‐metal active sites and diverse bonding energies, ensures intermediates are guided through energetically favorable pathways.

### 2.5 Unveiling Fe as a Hydrogen Shuttle Hub in HELMA

DFT modeling and reaction pathway analysis reveals that while Zn, Sn, Ni, and Bi serve as active catalytic sites throughout multiple steps of the NO_x_RR reaction, Fe does not directly interact with nitrogen intermediates as an active site. This raised the question of why the DoE included Fe in the final composition. Fe was among the top five elements identified due to its significant positive impact on electrochemical performance, suggesting it may play an indirect or supportive role in the electroreduction process. To clarify Fe's role, we investigated its involvement in the parallel hydrogen donation reaction. Fe uniquely exhibits negative free energy of hydrogen,^[^ ^38^, ^39^, ^40^ ^]^ indicating that it likely facilitates hydrogen supply to the reaction rather than directly bonding with nitrogen intermediates. However, a potential concern arises: hydrogen could saturate the Fe surface, potentially hindering its interaction with nitrogen or leading to hydrogen gas evolution.

**Figure 5:**
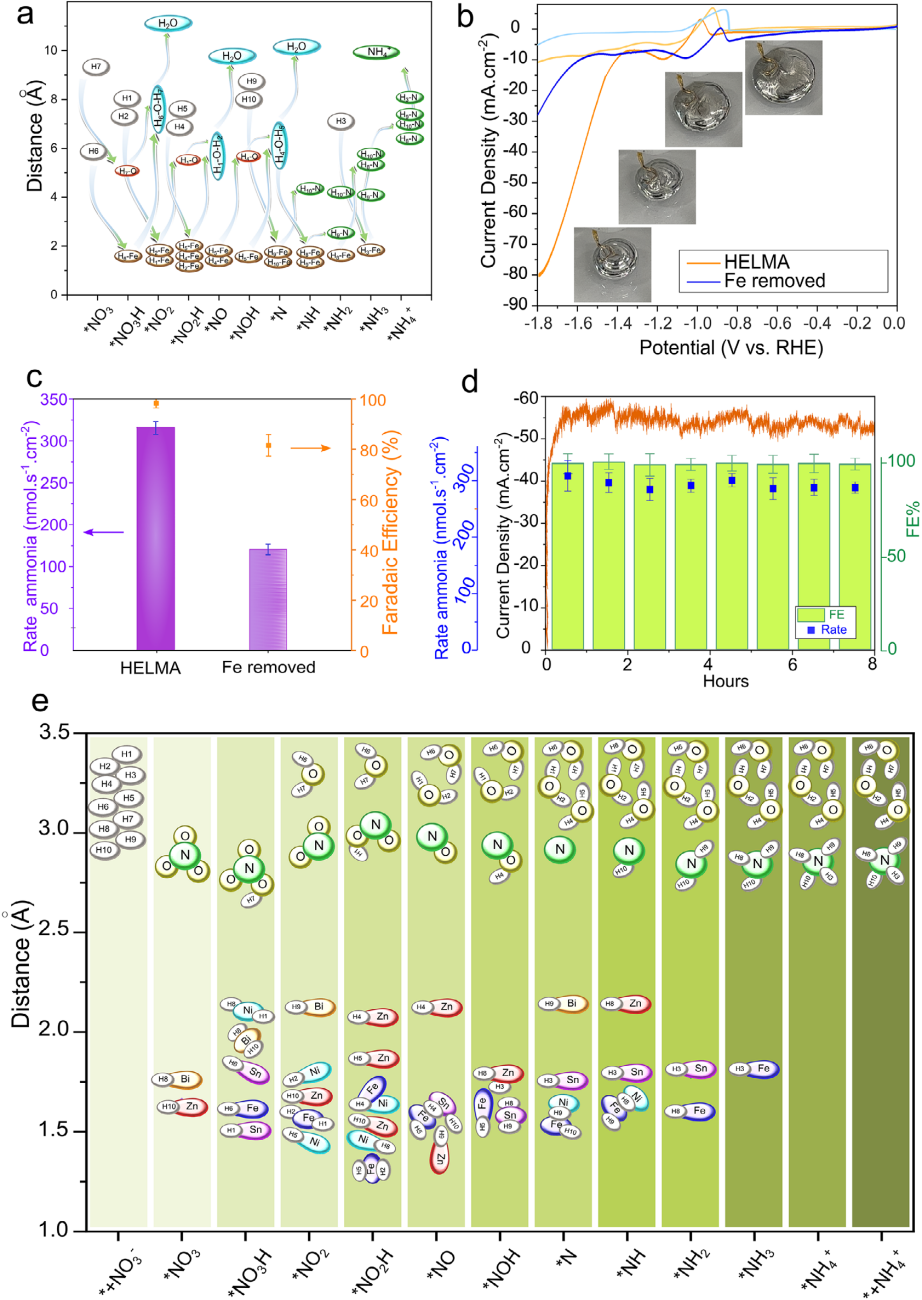
Experimental and computational evidence for Fe as a hydrogen shuttle hub in HELMA. DFT‐tracked trajectories of ten hydrogen atoms (H_1_–H_10_) relative to Fe throughout the NO_x_RR pathway, with arrows indicating sequential hydrogen transfers and reaction steps. b) LSV of HELMA and Fe‐removed HELMA in the blank and nitrate‐containing electrolyte; the inset displays morphological changes in the liquid alloy catalyst as the potential increases. c) Comparison of FE and ammonia production rate for HELMA with and without Fe, showing the critical role of Fe in both activity and selectivity. d) Chronoamperometric stability test over 8 h demonstrating the durability of HELMA with retained current density, FE, and production rate, enabled by sustained hydrogen shuttling through the Fe‐centered network. e) Time‐resolved distances between each hydrogen atom and all metal atoms across the full reaction sequence; only interactions within 2.2 Å are shown, highlighting the dynamic role of Fe, Sn, Ni, and Zn in hydrogen coordination and transfer.

We performed further DFT calculations to track hydrogen interactions with all metal atoms in the HELMA and understand how Fe and other elements contribute to hydrogen donation without impeding the reaction. We monitored ten hydrogen atoms (randomly labeled as H_1_–H_10_) and their distances to Fe and other metals during each reaction step. **Figure** **5**a shows hydrogen distances and their trajectories relative to Fe in the NO_x_RR. Initially, H_7_ interacts with the O─N bond, while H_6_ bonds with Fe and is eliminated as the first water molecule. Subsequently, H_1_ and H_2_ form a second water molecule, and H_4_ and H_5_ form a third. Fe continues to donate hydrogen atoms by sequentially shuttling H_8_, H_9_, and H_10_ to bond with ^*^N. Finally, H_3_ provides the last hydrogen to ^*^NH₃, leading to the formation of NH₄⁺.

To translate the atomistic picture from DFT into measurable electrocatalytic behavior, we prepared a control alloy in which Fe was selectively omitted while keeping the concentrations of Bi, Ni, Zn, and Sn unchanged (“Fe removed”). LSV in nitrate electrolyte (Figure 5b) reveals an immediate, three‐fold performance penalty: at −1.80 V versus RHE, the full HELMA reaches 80 mA cm⁻^2^, whereas the Fe‐free analogue plateaus at only 27 mA cm⁻^2^. The onset for cathodic current is also shifted by ≈120 mV to more negative potentials, evidencing a higher kinetic barrier when the Fe‐mediated proton pool is removed. Please note that a quick droplet shape changes and rapid droplet movement (Figure 5b‐inset), caused by surface tension variations due to changes in surface charge under applied cathodic current, led to a characteristic noise observed in all samples ≈−0.9 V versus RHE.

These current–density losses translate directly into diminished reaction metrics (Figure 5c). With Fe present, the ammonia production rate attains 320 nmol s⁻¹ cm⁻^2^ at ≈100% Faradaic efficiency; eliminating Fe cuts the rate to 150 nmol s⁻¹ cm⁻^2^ and the efficiency to 81%. The parallel drop in activity and selectivity is consistent with our computational finding that Fe binds H *just strongly enough* (ΔG_H^*^ < 0) to act as a relay, but not so strongly that it fosters parasitic H₂ evolution. When Fe is absent, protons arrive at the nitrate‐bound sites more slowly and less synchronously, allowing competitive pathways (hydrogen evolution, nitrite release) to reclaim charge and lower FE. Enhanced proton availability also manifests in durability. Under galvanostatic operation for 8 h (−50 mA cm⁻^2^, Figure 5d), the full HELMA maintains stable current, production rate, and FE. The data indicate that the dynamic “hydrogen circuit” centred on Fe accelerates the NO_x_RR cascade via HELMA and prevents local H‐oversaturation or catalyst poisoning, preserving the liquid alloy's reactive configuration over extended operation.

Figure 5e generalizes the single‐element insight to the full, five‐component liquid alloy. We uncover a hierarchical proton‐distribution network by mapping the time‐resolved distance of each of the ten tracked H atoms to every metal center. Fe remains the obligatory gatekeeper, but Sn, Ni, and Zn act as transient way‐stations that buffer and funnel hydrogen toward the reaction locus. At the same time, Bi stabilizes nitrogen‐derived intermediates without directly adsorbing H itself.

During the early O‐abstraction steps, H_6_ adsorbs on Fe and is delivered directly to the ^*^NO_x_ fragment, confirming Fe's kinetic priority. In parallel, H_1_ oscillates between Sn and Ni before passing to Fe, illustrating how moderate‐binding metals accumulate “stand‐by” protons that can be dispatched when a Fe site becomes vacant. Once the surface is stripped of oxygen, Bi locks the residual ^*^N in place, yet remains hydrogen‐repellent; H_10_, therefore, attaches directly to the Bi‐bound ^*^N, whereas H_8_ and H_9_ traverse Zn → Sn/Ni → Fe before reaching the same N center. The most extended excursion is traced by H3 (Zn → Sn → Fe → ^*^N), underscoring the fluid alloy's ability to reroute protons whenever local congestion develops.

This relay scheme overcomes the two main rate limitations of NO_x_RR: slow proton supply and site blocking by adsorbed H₂. Excess hydrogen can diffuse laterally onto Sn, Ni, or Zn instead of evolving as gas. Every proton eventually converges on Fe, just in time for the next hydrogenation step, ensuring synchronous electron‐proton transfer and maintaining near‐quantitative Faradaic efficiency over extended operation (Figure 5d). The significant activity loss observed for the Fe‐removed control (Figure 5b,c) reflects the failure of this self‐regulating hydrogen circuit: the auxiliary metals can still store protons, but without the Fe hub, they cannot discharge them efficiently to the nitrogen center.

Beyond mechanistic insight, these findings suggest a broader design principle for high‐entropy electrocatalysts. Combining a single “hub” metal with marginal‐binding “relay” elements and a strongly N‐affine “anchor” increases proton accessibility while maintaining selectivity. In HELMA, the liquid matrix entropically stabilizes this multi‐node network, allowing for rapid, reversible hydrogen migration while maintaining the high activity, selectivity, and durability shown in our research. To further improve metal utilization efficiency and active site exposure, future efforts will focus on depositing the liquid alloy as thin films or patterned microdroplets on conductive supports, thereby maximizing surface accessibility while retaining the advantages of the dynamic liquid interface.

## 3 Conclusion

In conclusion, we have developed an optimized high‐entropy liquid‐state catalyst that offers a barrier‐free electrocatalytic pathway for ammonia synthesis. Characterizing liquid metals is inherently challenging due to their opacity and lack of ordered structure, limiting the applicability of traditional techniques like electron and X‐ray diffraction. To overcome this, we combined experimental efforts with computational tools: MD simulations and DoE guided the selection of alloy compositions that remain liquid near room temperature while minimizing the number of experiments needed. DFT modeling provided critical insight into the fundamental catalytic activity. This integrated strategy not only informs catalyst design and mechanistic understanding but also leverages high configurational entropy to achieve atomically dispersed active sites without phase separation. DFT modeling of liquid high‐entropy catalysts proved essential for understanding this dynamic landscape, revealing a cooperative, multi‐element mechanism that enables energy barrier‐free ammonia synthesis. These insights highlight the potential of tailored high‐entropy liquid catalysts for efficient and scalable ammonia electrosynthesis, advancing sustainable energy solutions. By integrating high configurational entropy, hydrogen shuttling, dynamic sites, and barrier‐free interactions, this work establishes a foundation for one‐piece atomic “handshakes,” creating a highly reactive platform for complex chemical transformations.

Our alloying strategy achieved atomic dispersion and uniformity in the liquid state, isolating high‐entropy effects for precise catalyst design. The liquid metal matrix fosters a dynamic environment, enabling intermediates to interact with various active sites and improving reaction efficiency. The cooperative interplay among the active elements ensures efficient nitrate deoxygenation, nitrogen hydrogenation, and hydrogen management. Fe emerges as the primary site for hydrogen activation, while other elements prevent surface saturation and hydrogen gas evolution, thereby sustaining catalytic activity. These findings demonstrate how the structural flexibility and lack of short‐range order in high‐entropy materials enhance their catalytic properties. The observed dynamic atomic diffusion in our liquid‐phase system exemplifies the predicted nanoscale structural adaptability of HEAs, delivering a unique catalytic environment unattainable in solid‐state systems.

## 4 Experimental Section

### Synthesis of the Catalysts—Materials

Gallium beads, sourced from Indium Corporation (USA), in their 99.999% pure form, and bismuth ingots, obtained from Rotometals (USA), in their 99.999% pure form, all other metals obtained from Sigma–Aldrich in their 99.999% purification, were used as received for alloy preparation.

### Synthesis of the Catalysts—Alloy Preparation

Alloys were synthesized by initially heating a mixture of gallium beads (99.99% purity, Indium Corporation) and the desired metals listed in Table S1 (Supporting Information), sourced from Sigma–Aldrich and Indium Corporation. Binary alloys were prepared first, and the additional compositions in Table S1 (Supporting Information) were designed based on a planned experimental design discussed in subsequent sections. The mixtures were formulated according to phase diagrams and eutectic ratios, maintaining a composition of 100 % minus the weight percent of additional metals, with each additional metal at 0.5 wt.%. The prepared mixture underwent high‐temperature treatment at 1500 °C in an argon‐filled furnace for 3 h to initiate alloying.

A post‐heating mechanical grinding process incorporated metals with higher melting points into the liquid gallium matrix. After the initial heating phase, the mixture was cooled to 80 °C, at which point the liquid alloy, potentially containing clusters of higher melting point metals, was carefully transferred to a mortar. In a controlled glovebox environment filled with inert gas to prevent oxidation and contamination, the alloy was ground and mixed incrementally, reducing the temperature by 10 °C intervals to 40 °C. Each mixing step involved 10 min of manual grinding using a mortar and pestle. This iterative process continued until the alloy reached near room temperature. Mechanical grinding was essential for achieving two primary objectives: removing surface oxides and thoroughly homogenizing the constituent metals. This meticulously controlled procedure resulted in a uniformly atomically dispersed, multi‐component liquid metal alloy with enhanced homogeneity and stability.

### Synthesis of Ammonia—Reagents and Materials

All necessary reagents and solvents were procured from Sigma–Aldrich or Chem‐Supply Pty Ltd.

### Synthesis of Ammonia—Electrochemical Measurement and Evaluation

The electrochemical evaluations were conducted using a CHI650E potentiostat (CH Instruments Inc., USA) in conjunction with a single‐chamber cell. The working electrode (WE) utilized a liquid alloy catalyst. A platinum mesh served as the counter electrode (CE), while the reference electrode (RE) was an Ag/AgCl electrode in a saturated KCl solution. The background electrolyte consisted of 0.5 m Na2SO4 in 10 mm H2SO4, with the acid concentration optimized accordingly. The electrochemical cell had a volume of 20 mL, and the electrode volume was 40 µL, with an active surface of 0.565 cm^2^. Pure Ga and HELMA without Fe were employed as control samples, and a magnetic stirrer was set at 500 rpm to facilitate the reaction. All experiments were conducted on a hot plate at a temperature of 40 °C.

Ammonia (as ammonium NH4+) detection by the indophenol blue method. The 0.5 mL electrolyte sample was transferred to a 2 mL sample tube. To the tube, 0.4 mL of 1 m NaOH solution (containing 5 wt.% salicylic acid and 5 wt.% sodium citrate), 0.1 mL of 0.05 m NaClO, and 30 µL of 1 wt.% C5FeN6Na2O (sodium nitroferricyanide) in water was added. The mixture was then incubated in the dark at room temperature for 2 h before UV–vis testing. A calibration curve was prepared using standard (NH4)2SO4 solutions in 10 mm H_2_SO_4_ with the indophenol blue reagents mentioned above to determine the ammonia concentration. The absorbance of the indophenol blue was measured at 655 nm after 2 h. The detection limit for UV–vis analysis was defined as the absorbance at 655 nm, with the lower limit set as the blank 0.5 m Na_2_SO_4_ in 10 mm H_2_SO_4_ and the upper limit as 200 µm NH_4_^+^ (Figure S9, Supporting Information).

### Synthesis of Ammonia—Ammonia Detection

The performance of ammonia synthesis was evaluated based on two key indicators: Faradaic efficiency and ammonia production rate. Faradaic efficiency measures the selectivity of the electrocatalyst for ammonia synthesis. It was determined by the ratio of electrical energy consumed for ammonia synthesis to the total energy in the electrochemical system. It can be calculated using the following equation:

(3) $$\eta =\frac{n\cdot F\cdot C\cdot V}{Q}$$

In the equation, n represents the number of electrons required for synthesizing one ammonia molecule (*n* = 8 for nitrate), F denotes the Faraday constant (96 485.33 C mol^−1^), C signifies the detected ammonia molar concentration, V represents the volume of the electrolyte, and Q corresponds to the total electrical energy applied to the electrodes. The number of electrons exchanged was determined by measuring the nitrate concentrations before and after each reaction.

The rate of ammonia production (R) was determined by dividing the amount of ammonia produced by the time elapsed and the electrode surface area. The formula for calculating R is as follows:

(4) $$R=\frac{C\cdot V}{t\cdot S}$$

In the equation, C represents the detected ammonia molar concentration, V denotes the electrolyte volume, t signifies the reaction time, and S corresponds to the surface area of the catalytically active electrode.

### Characterization

In this study, the morphology and elemental distribution of the samples were characterized using field‐emission SEM (Nova Nano SEM) and TEM (JEOL JEM‐F200) equipped with an EDS detector and a GATAN ORIUS camera. Direct digital imaging was used for morphological and structural analysis. To prepare the samples for SEM imaging (Figure 1e–h), a touch print was made on silicon wafers, attached them to double‐sided carbon tape, and loaded them into the SEM machine. To prepare the samples for SEM imaging (Figure 3f ii–iii), nitrogen gas was used to freeze the catalyst after the reaction, attached them to double‐sided carbon tape, and loaded them into the SEM machine. A touch print was made onto a lacey carbon grid to prepare TEM samples and loaded into the TEM instrument using a double‐tilt holder. The XRD patterns were obtained using an expert Multipurpose X‐ray diffraction system (PANalytical, with a wavelength of 1.54 Å and Cu Kα radiation) to obtain structural characterization. The parameters for the acquisition were: voltage of 45 kV, current of 40 mA, 2θ range from 10° to 70°, time pre‐step of 120 s, and a step size of 0.026°. A Netzsch DSC 204 F1 instrument was utilized for the DSC measurements. XPS was conducted using a Thermo Scientific K‐Alpha XPS spectrometer featuring a monochromated Al Kα X‐ray source with a photon energy of 1486.7 eV and an X‐ray spot size of 30–400 µm. To characterize the HELMA via TEM, a fine layer was transferred via touch printing onto a TEM grid (Lacey carbon grids, Prositech). TEM imaging was conducted using a JEOL 2100F TEM/STEM instrument equipped with a Gatan OneView 4k CCD Camera. Measurements were performed with a 200 keV acceleration voltage.

### Design of Experiments

In this study, DoE methodology was employed to optimize HELMA catalysts for NOxRR, targeting high ammonia production rate, Faradaic efficiency, and ammonia concentration. A face‐centered central composite design with three center‐point replicates was implemented using Design‐Expert software, evaluating 13 metals (Mo, Mn, Ru, Ag, Zn, In, Ni, Bi, Cu, Fe, Pd, Sn, and W) as variables, with gallium as the primary component to maintain the liquid phase. These metals were selected based on their reported efficacy in nitrate reduction. The DoE framework generated 28 unique alloy compositions, which were experimentally tested for their responses (ammonia concentration, rate, and Faradaic efficiency). Experimental data were fitted to a multiple non‐linear regression model, capturing up to three‐way interactions between elemental compositions:

(5) $$\begin{array}{ccc} R_{1} & = & 94.81+2.47\mathrm{Mn}+4.2\mathrm{Ag}-5.52\mathrm{In}+5.4\mathrm{Ni}+9.2\mathrm{Bi}-6.17\mathrm{Pd}\\ & & +6.14\mathrm{Sn}-11.44\mathrm{MoZn}-1.44\mathrm{MnSn}+17.38\mathrm{AgIn}+3.42\mathrm{AgW}\\ & & +3.79\mathrm{ZnNi}+6.71\mathrm{ZnFe}-0.13\mathrm{ZnW}-0.17\mathrm{InW}+0.14\mathrm{NiFeW}\\ & & +0.09\mathrm{CuPdW} \end{array}$$

(6) $$\begin{array}{ccc} R_{2} & = & 94.9-23.4\mathrm{Mo}-9.45\mathrm{Ag}+8.12\mathrm{Ni}+19.7\mathrm{Bi}+3.45\mathrm{Cu}-9.88\mathrm{Pd}\\ & & +2.54\mathrm{MoBi}-1.48\mathrm{MnFe}+8.34\mathrm{MnPd}-9.19\mathrm{AgZn}-2.12\mathrm{ZnFe}\\ & & -7.65\mathrm{InSn}-3.51\mathrm{InW}-9.89\mathrm{CuSn}+0.33\mathrm{RuAgW}+0.05\mathrm{AgFeSn}\\ & & +0.06\mathrm{InNiCu} \end{array}$$

(7) $$\begin{array}{ccc} R_{3} & = & 277.8+7.25\mathrm{Mn}+12.3\mathrm{Ag}-16.18\mathrm{In}+15.82\mathrm{Ni}+26.97\mathrm{Bi}\\ & & -18.07\mathrm{Pd}+17.99\mathrm{Sn}-23.5\mathrm{MoZn}-10.03\mathrm{MoNi}-3.35\mathrm{MnSn}\\ & & +40.89\mathrm{AgIn}+21.13\mathrm{ZnNi}+19.67\mathrm{ZnFe}-28.48\mathrm{ZnW}-17.87\mathrm{InW}\\ & & +0.23\mathrm{NiFeW}+0.12\mathrm{CuPdW} \end{array}$$

Statistically insignificant variables were discarded, and interactions were analyzed to identify synergistic effects and prevent phase segregation due to atomic radii mismatches. DoE along with MD gives the optimal composition, Ga (97.5 wt.%) with Fe (0.5 wt.%), Bi (0.5 wt.%), Ni (0.5 wt.%), Zn (0.5 wt.%), and Sn (0.5 wt.%), was determined to balance performance and stability, as detailed in Figure 4a.

### Molecular Dynamic Method

In this study, MD simulations were performed to estimate the composition of liquid metal alloys and determine the maximum concentration of secondary metals that remain liquid near room temperature of 400 atoms in a 22.2 × 38.0 × 9.1 Å^3^ box ab initio MD simulations were carried out in the Vienna ab initio Simulation Package at the desired temperature using the projector‐augmented wave method, the PBE exchange‐correlation functional, an energy cut‐off of 350 eV, and a 1 × 1 × 2 k‐point grid. The atomic arrangements were examined using the radial distribution function (RDF), g(r), which provides a statistical measure of atomic packing and was invaluable for probing short‐ and medium‐range order. Specifically, second‐nearest‐neighbor (2NN) correlations emerged as a critical feature influencing the structural transitions during liquefaction.

### Density Function Theory Methods

This work employed the plane‐wave VASP code for all DFT calculations.^[^ ^42^, ^43^, ^44^ ^]^ The core electrons in our calculations were treated using the projector augmented wave method, and the Perdew–Burke–Ernzerhof exchange‐correlation functional^[^ ^45^ ^]^ was employed. The cut‐off value for the wavefunctions was set to 500 eV, and a gamma‐point k‐grid was used. Relaxation calculations ended when the residual forces on atoms were less than 0.03 eV Å^−1^. A vacuum region greater than 10 Å in the direction normal to the sheets was used to avoid interactions between periodic images.

Mechanistic investigations of NO_x_RR and HER were performed on a liquid gallium‐based alloy (including Mo, Mn, Ru, Ag, Zn, In, Ni, Bi, Cu, Fe, Pd, Sn, and W) slab which contained 32 atoms (a = 8.86 Å, b = 15.19 Å, c = 14.55 Å, 𝛼 = 90.0^°^, 𝛽 = 90.0^°^, 𝛾 = 90.0^°^, where c was the surface normal direction). In the first step, binary alloys of Ga and second metal were created by doping one of the surface Ga atoms with the second metal's atom to investigate the effect of defects on the catalytic processes. The below steps of NO_x_RR were computed for pure Ga, 13 binary alloys, and HELMA (Equations 1 and (8), (9), (10), (11), (12), (13), (14), (15), (16), (17), (18), (19)).

(8) $$*+{\mathrm{NO}}_{3}^{-}\to {\mathrm{NO}}_{3}^{*}+e^{-}$$

(9) $${\mathrm{NO}}_{3}^{*}+H^{+}+e^{-}\to NO_{3}H^{*}$$

(10) $$NO_{3}H^{*}+H^{+}+e^{-}\to {\mathrm{NO}}_{2}^{*}+H_{2}O$$

(11) $${\mathrm{NO}}_{2}^{*}+H^{+}+e^{-}\to NO_{2}H^{*}$$

(12) $$NO_{2}H^{*}+H^{+}+e^{-}\to NO^{*}+H_{2}O$$

(13) $$NO^{*}+H^{+}+e^{-}\to \mathrm{NO}H^{*}$$

(14) $$\mathrm{NO}H^{*}+H^{+}+e^{-}\to N^{*}+H_{2}O$$

(15) $$N^{*}+H^{+}+e^{-}\to NH^{*}$$

(16) $$NH^{*}+H^{+}+e^{-}\to {\mathrm{NH}}_{2}^{*}$$

(17) $${\mathrm{NH}}_{2}^{*}+H^{+}+e^{-}\to {\mathrm{NH}}_{3}^{*}$$

(18) $${\mathrm{NH}}_{3}^{*}+H^{+}\to {\mathrm{NH}}_{4}^{+*}$$

(19) $${\mathrm{NH}}_{4}^{+*}\to *+{\mathrm{NH}}_{4}^{+}$$

The free energy changes were computed using the computational hydrogen electrode approach,^[^ ^46^ ^]^ where the free energy equals a standard hydrogen electrode. The free energy of (𝐻^+^ + 𝑒^−^) equals 1/2H_2_(g) various intermediates is calculated as:

(20) $$G=E_{elec}+E_{ZPE}-TS$$

where E_elec_, is the total electronic energy computed by DFT, EZPE is the zero‐point energy, T is temperature, and 𝑆 is entropy. For H adsorption, the change in free energy is computed as eV, ∆𝐺 = ∆𝐸_𝑒𝑙𝑒𝑐_ + 0.24 eV, where the value 0.24 eV, taken from refs. [5, 45] includes the corrections to the ZPE and the entropy terms. For the NO_x_RR intermediates, the correction terms were neglected – this, however, does not affect the key conclusions drawn from our DFT calculations.

## Conflict of Interest

The authors declare no conflict of interest.

## Author Contributions

Conceptualization was carried out by S.N. and A.R.J. Methodology was developed by S.N., A.N., P.V.K, D.E., and A.R.J. Investigation was conducted by S.N. and A.N. Visualization was prepared by S.N. and A.R.J. Funding acquisition was secured by P.V.K, D.E., and A.R.J. Project administration and supervision were managed by P.V.K, D.E., and A.R.J. Writing of the original draft was done by S.N. and A.R.J. Writing, reviewing and editing were performed by A.N., P.V.K, A.Z., A.R.N., F.M.A., and D.E.

## Supporting information



Supporting Information

Supplemental Movie 1

## Data Availability

The data that support the findings of this study are available from the corresponding author upon reasonable request.
