# Supplementary material for: Configuring a Liquid State High‐Entropy Metal Alloy Electrocatalyst
Source: Small. 2025 Jun 17;21(32):2504087. doi: 10.1002/smll.202504087 (PMC12366271; doi:10.1002/smll.202504087)
Supplement: Supplementary file 1 — Supporting Information [file SMLL-21-2504087-s002.docx]

Supplementary Information

**Supplementary Text**

**Molecular dynamics (MD) simulations**

**Radial Distribution Function (RDF), g(r).**

Regarding MD simulations and RDF analysis of gallium-based liquid metal alloys, RDF exhibits multiple peaks, with the sharpness and position of these peaks encoding atomic order. The first peak corresponds to the nearest-neighbor shell, reflecting the immediate environment around a reference atom. However, the second peak in the RDF is particularly insightful as it arises from the 2NN shell and reveals the connectivity of atomic packing motifs. As shown in a recent work,^1^ the 2NN correlations are strongly influenced by the type of shared atomic connections—vertex-sharing (1-atom), edge-sharing (2-atom), face-sharing (3-atom), and distorted tetrahedra (4-atom) connections.

Figure S 2 shows the RDF of alloy concentrations exhibiting sharp peaks at lower temperatures due to rigid atomic positions consistent with ordered structures or glassy states. As temperature increases, the RDF peaks broaden and diminish, reflecting the collapse of higher-order atomic correlations and increasing atomic mobility. In binary gallium-based alloys, the RDF’s higher-order peaks collapsed near room temperature (30^°^C to 40^°^C) for secondary metal concentrations up to 0.5 wt%. Beyond this threshold, residual peaks persisted, indicating partial solidification and residual medium-range order.

The persistence of these peaks can be linked to the preferential face-sharing (3-atom connections) observed in many metallic glasses and liquids. During cooling, face-sharing becomes dominant, replacing weaker edge- and vertex-sharing connections. This trend enhances local rigidity and inhibits complete liquefaction at higher concentrations. Notably, face-sharing connections stiffen the elastic response of atomic clusters, as evidenced by quasi-static shear deformation simulations, which showed minimal shear strain for face-sharing clusters compared to more flexible 2-atom and 4-atom configurations.

Splitting of the second RDF peak, often observed in metallic systems, also played a role in identifying structural transitions. While peak splitting was traditionally attributed to glassy ordering, recent findings suggest that it can emerge intrinsically from cluster connections even in equilibrium liquids. For alloys with high atomic similarity, the uneven contributions of face- and edge-sharing clusters can generate a split-second peak, serving as a structural fingerprint for the underlying medium-range order.

Ultimately, by analyzing the evolution of the RDF as a function of temperature and composition and leveraging 2NN correlations, we pinpointed the conditions under which medium-range order diminishes, and alloys transition into a fully liquid state. These findings were reinforced by complementary MD simulations, DoE, and DFT analyses, which revealed that high configurational entropy prevents phase separation and maintains uniform active sites. This atomic-scale understanding of liquid alloy structure offers a precise roadmap for designing alloys with tailored liquidity, which is critical for catalytic and other advanced applications.

**Segmented regression analysis**

One of the key innovations of this study is the use of MD simulations to predict the melting behavior of high-entropy liquid metal alloys, enabling the precise identification of compositions that remain liquid near room temperature. By modeling the thermodynamic properties of various alloy compositions, we established a feedback loop between computational predictions and experimental synthesis. This computational guidance allowed us to pinpoint the optimal concentrations of alloying elements necessary to stabilize the liquid phase, significantly reducing the experimental workload required to produce these advanced materials.

To investigate both liquid and solid phases in binary and HELMA alloys, MD simulations were conducted across a temperature range of 273 K to 573 K, maintaining the same experimental composition ratio. The primary structure consisted of 400 atoms, initially considered as solid. This structure transitioned to a semiliquid state and eventually to a liquid phase at higher temperatures.

We employed the Maximum-Minimum Distance Selection algorithm for the initial atomic distribution in the primary structure. This algorithm addresses a diversification problem by selecting a subset of points in a 3D space to maximize the minimum pairwise distance. Using a greedy iterative approach, the method ensures computational efficiency while achieving high-quality solutions for atomic placement.

We performed MD simulations across a range of concentrations (0.25, 0.5, 0.75, 1, and 1.5 wt%) to determine the maximum achievable concentration of secondary elements capable of remaining liquid at near-ambient conditions. These simulations identified 0.5 wt% as the upper limit for ensuring that binary alloys and HELMAs retain their liquid state near room temperature. By providing a clear compositional target, the MD results minimized the number of trial-and-error experiments needed, streamlining our search for stable liquid alloys.

This strategy addresses two central challenges. First, it ensures the thermodynamic stability of HELMAs, enabling them to maintain a liquid state under near-ambient conditions, a requirement critical for their catalytic applications. Second, it improves experimental efficiency by guiding synthesis efforts toward compositions predicted to exhibit the desired phase behavior, thus minimizing trial-and-error approaches.

The energy-temperature relationship, derived from the MD simulations, is depicted in Figure S 5. The plot reveals a significant structural transition as temperature increases, characterized by a phase change from solid to liquid. To delineate the intermediate semiliquid phase, piecewise linear regression was applied to segment the energy-temperature data into distinct regions. This approach allowed us to identify the temperature range where the material exhibits semiliquid characteristics, providing critical insights into the experimental condition selection. Figure S 6, the HELMA alloy transitions to a complete solid phase at 304 K. See also Figure S 3ci, which shows the segmented regression analysis for binary alloys containing 0.5 wt% metal, derived from MD simulations over a broad temperature range and extracted from this data. To ensure the alloy remains entirely in the liquid phase, all ammonia synthesis procedures were conducted at 313 K.

**Design of experiments (DoE)**

Effective NO_x_RR electrocatalysts for ammonia synthesis require properties such as high activity for nitrogen species adsorption and conversion, selectivity for ammonia production, and stability under reaction conditions. These electrocatalysts must efficiently facilitate the adsorption of NO_x_ intermediates (nitrate, nitrite, or nitric oxide) while avoiding competing hydrogen evolution reactions. Key factors influencing the performance include catalyst material, surface structure, and dynamic adaptability to promote multi-step proton-coupled electron transfer pathways while maintaining optimal binding energy for intermediates. Implementing the DoE methodology in this study marks a significant advancement in catalyst alloy design for electrochemical ammonia synthesis. Unlike conventional one-variable-at-a-time approaches, which oversimplify the complex relationships within multivariable systems, DoE provides a systematic and efficient framework for exploring and optimizing experimental conditions. By simultaneously evaluating all experimental factors, DoE ensures that individual effects and critical multi-way interactions are captured. This approach minimizes the number of experimental runs while providing deeper insights into the influence of various metal elements on key performance metrics such as ammonia production rate, Faradaic efficiency, and ammonia concentration.

One of the primary strengths of DoE lies in its ability to uncover non-linear relationships and synergistic interactions between multiple variables. Unlike traditional methods that assume additive effects and linear behavior, DoE allows for identifying complex relationships that govern catalyst performance. This study highlights the importance of collective contributions over isolated effects by analyzing interactions between metal elements. Statistically insignificant variables are systematically discarded, allowing the focus to remain on impactful factors. Furthermore, statistical tools, such as regression models, provide predictive capabilities that enable the identification of optimal alloy compositions. This ensures that the optimized conditions reflect the global optima of the system rather than being constrained to local solutions, a frequent limitation of one-variable-at-a-time approaches.

The DoE framework proves particularly valuable in designing high-entropy liquid metal alloys, where the interplay between multiple metals determines performance and stability. In such systems, achieving homogeneity and preventing phase segregation are critical challenges, as atomic radii mismatches can lead to alloy instability. DoE addresses this challenge by systematically evaluating the role of each metal and its combinations, allowing for the identification of synergistic compositions that enhance catalytic efficiency. This process identifies an optimized alloy composition that successfully balances ammonia production rate, Faradaic efficiency, and ammonia concentration while ensuring long-term stability.

Gallium was selected as the primary component to ensure the alloy remains in the liquid phase. Thirteen metals (Mo, Mn, Ru, Ag, Zn, In, Ni, Bi, Cu, Fe, Pd, Sn, and W), previously reported in the literature for their high performance in nitrate reduction, were chosen as the initial elements for the DoE. These metals served as the primary variables in the DoE framework, while ammonia concentration, ammonia rate, and Faradaic efficiency were treated as the response variables. The goal was to maximize these response values while maintaining the durability of the liquid catalyst. A face-centered central composite design with three replicates at the center point was employed for the DoE. All mathematical analyses and experimental designs were conducted using Design-Expert software. This approach resulted in 28 unique LMAC compositions to be experimentally tested for their responses (Table S2). The experimental data were then fitted to a multiple non-linear regression model (Eq. SR1), enabling predicting responses (R) as functions of the elemental composition and their interactions, including up to three-way interactions. Subsequently, the contributions of individual elements and their interactions were analyzed chemically and physically. Metals with significantly different atomic radii were observed to induce liquid phase separation rather than forming a homogeneous substitutional and interstitial alloy. Based on these analyses, the optimal alloy composition was identified as Ga (97.5 wt%) combined with Fe (0.5 wt%), Bi (0.5 wt%), Ni (0.5 wt%), Zn (0.5 wt%), and Sn (0.5 wt%). This composition aligns with the criteria for high-entropy liquid metal alloys (HELMAs), which exhibit superior performance and stability. An overview of the discarded model coefficients and their corresponding elements for each response is provided in Figure 3a. Those elements not included in the table for each response are not recognized as statistically significant (their coefficients are estimated as zero).

$R=\beta_{0}+\sum_{i=1}^{13} \beta_{i}.{Element}_{i}+\sum_{i=1}^{13} \sum_{j=i+1}^{13} \beta_{ij}.({Element}_{i}\times{Element}_{j})+\ldots+ℇ$ S Eq 3

Where β_0_ is the constant of the fitted function, β_i_ are coefficients for the main effects, and β_ij_ are coefficients for the interaction effects between each pair of elements.

$$R_{1}=94.81+2.47Mn+4.2Ag-5.52In+5.4Ni+9.2Bi-6.17Pd+6.14Sn-11.44MoZn-1.44MnSn+17.38AgIn+3.42AgW+3.79ZnNi+6.71ZnFe-0.13ZnW-0.17InW+0.14NiFeW+0.09CuPdW$$

$$R_{2}=94.9-23.4Mo-9.45Ag+8.12Ni+19.7Bi+3.45Cu-9.88Pd+2.54MoBi-1.48MnFe+8.34MnPd-9.19AgZn-2.12ZnFe-7.65InSn-3.51InW-9.89CuSn+0.33RuAgW+0.05AgFeSn+0.06InNiCu$$

$$R_{3}=277.8+7.25Mn+12.3Ag-16.18In+15.82Ni+26.97Bi-18.07Pd+17.99Sn-23.5MoZn-10.03MoNi-3.35MnSn+40.89AgIn+21.13ZnNi+19.67ZnFe-28.48ZnW-17.87InW+0.23NiFeW+0.12CuPdW$$

By capturing non-linear relationships and up to three-way interactions between elemental compositions, this approach provides unprecedented insights into the synergistic roles of individual metals and their collective performance. Unlike traditional catalyst design, which often relies on trial-and-error methods, the DoE framework offers a data-driven strategy that accelerates the discovery of optimal alloy compositions. The study further pioneers the application of high-entropy effects within a liquid metal matrix, a novel platform that prevents phase segregation and ensures atomic-level dispersion of active metals. This dynamic system enables adaptable catalytic sites, enhancing nitrogen deoxygenation, hydrogenation, and hydrogen management efficiency. Combining experimental optimization with theoretical DFT modeling further refines catalyst performance by elucidating the synergistic contributions of specific elements, such as Fe’s role in hydrogen shuttling and Zn, Ni, Sn, and Bi’s participation in nitrogen reduction steps. Together, these innovations establish a robust, scalable framework for designing high-entropy liquid metal alloys with superior stability, performance, and versatility, paving the way for advanced catalytic systems in sustainable energy applications.

The predictive models developed using DoE are central in extending the study’s findings beyond the experimentally tested conditions. These models correlate elemental composition with catalyst performance, enabling the prediction of new compositions without the need for exhaustive experimental testing. The visual representation of results, such as through interaction plots, provides an intuitive understanding of the relationships between variables and their collective impact on performance. By integrating these models with theoretical insights, such as DFT analysis, the study offers a detailed understanding of the mechanistic roles played by individual metals in enhancing the nitrogen reduction process.

**DFT procedure**

DFT simulations were systematically employed to clarify the proposed nitrate reduction mechanism, following a multi-step strategy to pinpoint the optimal catalysts and pathways for nitrate reduction.

Step 1: Screening Binary Liquid Alloys

Thirteen binary alloys (metal + gallium) identified from the literature were studied to explore their potential in nitrate reduction. Despite the inherent higher entropy of binary liquid alloys, the analysis of both reaction pathway energies and hydrogen evolution reaction (HER) revealed that they do not necessarily outperform greatly (Figure 2d,e). Each element's distinct reaction pathways for nitrate reduction involve endothermic energy changes. These endothermic sections are thermodynamically unfavorable and act as barriers, reducing nitrate reduction efficiency (table S3). Such steps hinder the progression of the reaction, making it difficult for the system to achieve continuous and efficient catalytic activity. Also, for some elements, Sn, Zn, Ru, Bi, Mn, W, Ga, and Mo, the HER is highly exothermic, meaning these catalysts strongly bind hydrogen. This strong hydrogen binding saturates the catalytic surface with hydrogen atoms, effectively blocking active sites and preventing the adsorption and reduction of nitrate species. As a result, the catalyst becomes inactive for nitrate reduction, with HER dominating the catalytic process instead. This observation highlighted the need for a deeper examination of key elements contributing to nitrate reduction.

Step 2: Identifying the most favorable approaching head

DFT analysis and DoE insights identified Ni, Fe, Zn, Bi, and Sn as critical elements for NO_x_RR when combined with gallium. These elements were central in determining reaction pathways, as further simulations revealed their capacity to achieve minimum energy configurations. To assess the favorability of different approaching heads for nitrate reduction to ammonia, energy results from DFT calculations were analyzed across 13 reaction steps involving five binary liquid alloy catalysts with different adsorption configurations (Table S4). The most favorable configuration in each step was identified based on the minimum energy. The probabilistic analysis quantified NE's dominance—where nitrogen approaches the additional element in the binary alloy—emerging as the most energetically favorable pathway across multiple steps and catalysts, highlighting its consistent stability and efficiency in stabilizing intermediates and facilitating conversion processes.

Step 3: Optimizing Reaction Pathway for HELMA

To identify a reaction pathway that minimizes energy barriers and facilitates efficient ammonia synthesis using HELMA catalyst, we established two key criteria: first, the surface should favor nitrogen adsorption over hydrogen to prevent hydrogen saturation; second, at each reaction step, the metal providing the lowest free energy should be selected, provided it does not lead to significantly higher free energy in subsequent steps. In other words, which element of HELMA (Ni, Fe, Zn, Bi, and Sn) plays a critical role in each reaction step. A representative catalyst model was constructed to achieve this, incorporating a liquid gallium slab doped with the five elements in a high-entropy matrix. Five sets of experiments, including 13 steps of the reaction pathway, were conducted, and each set of calculations focused on one element at a time, dissecting its specific role and impact on the reaction mechanism. Detailed DFT simulations involving 13 distinct steps of NO_x_RR were conducted for this complex alloy (Figure S 7).

Step 4: Balancing Energetics and Performance

The selection criteria for the critical metal at each reaction step extended beyond the sole minimization of energy levels. The analysis incorporated key considerations such as hydrogen evolution reaction (HER) efficiency and the avoidance of endothermic pathways. The SEq 5-12 conditions guided the optimization process, aiming to maximize or minimize the absolute differences between consecutive energy values, as dictated by specific energy-level constraints. The equations provide a systematic framework for optimizing reaction pathways by determining the optimal X_n_, ensuring the most efficient progression of the reaction sequence.

$if \left| X_{n} \right|>\left| X_{n-1} \right| then \left| X_{n} \right|-\left| X_{n-1} \right|=Max$ S Eq 5

$if \left| X_{n+1} \right|>\left| X_{n} \right| then \left| X_{n+1} \right|-\left| X_{n} \right|=Max$ S Eq 6

$if \left| X_{n} \right|>\left| X_{n-1} \right| then \left| X_{n} \right|-\left| X_{n-1} \right|=Max$ S Eq 7

$if \left| X_{n+1} \right|<\left| X_{n} \right| then \left| X_{n} \right|-\left| X_{n+1} \right|=Min$ S Eq 8

$if \left| X_{n} \right|<\left| X_{n-1} \right| then \left| X_{n-1} \right|-\left| X_{n} \right|=Min$ S Eq 9

$if \left| X_{n+1} \right|>\left| X_{n} \right| then \left| X_{n+1} \right|-\left| X_{n} \right|=Max$ S Eq 10

$if \left| X_{n} \right|<\left| X_{n-1} \right| then \left| X_{n-1} \right|-\left| X_{n} \right|=Min$ S Eq 11

$if \left| X_{n+1} \right|<\left| X_{n} \right| then \left| X_{n} \right|-\left| X_{n+1} \right|=Min$ S Eq 12

Step 5: Hydrogen trajectory analysis

We conducted DFT modeling to track the interactions between hydrogen atoms and all metal atoms within the HELMA to understand better how Fe and other elements contribute to hydrogen donation without hindering the reaction. We randomly numbered all ten hydrogen atoms as H_1_ to H_10_ and monitored their distances to Fe and the other metals during each reaction step and intermediate formation. A distance of less than 2.2 Å was considered indicative of bond formation. Figure S 8 shows the calculated distances between hydrogen atoms and each metal across all 13 steps. The first hydrogen (H_7_) directly interacted with O-N; subsequently, all other hydrogen radicals first adsorbed onto Fe. As shown in Figure S 8, the first water molecule forms while H_6_ bonds with Fe and H_7_ with oxygen. Subsequently, H_1_ and H_2_ bond with Fe to form the second water molecule, followed by H_4_ and H_5_, forming the third water molecule. Fe continues to donate hydrogen atoms, with H_10_ directly interacting with *N, followed by H_9_ and H_8_. H_3_ is the last hydrogen Fe provides to *NH₃ before the product departs as NH₄⁺.

Furthermore, we found that other metals also play significant roles in hydrogen interactions. H_6_ first approaches to directly bond with Fe. In parallel, H_1_ initially binds to Sn and Ni before transferring to Fe once H_6_ vacates the Fe surface. Similar processes occur during the elimination of the second and third oxygens, where hydrogen atoms shuttle between Sn, Ni, Zn, and Fe. This pattern highlights the conclusion that one hydrogen radical attaches directly to Fe while another is reserved with other metals. When the first hydrogen attaches to oxygen and departs as part of a water molecule, the second hydrogen finds Fe free, bonds to it, and then joins with oxygen to form another water molecule. This dynamic shuttling ensures efficient hydrogen donation and prevents hydrogen saturation on Fe, thereby facilitating the formation of water molecules without hindering nitrogen reduction. Similar processes occur with all subsequent oxygens, and a comparable mechanism is observed during the hydrogenation of N.

After eliminating all oxygen atoms, *N remains on the surface attached to Bi. Then, H_10_, directly bonded with Fe, acts as the first hydrogen attaching to the *N. During the second and third hydrogenation steps, Sn, Zn, and Ni facilitate the transfer of H_8_ and H_9_ to Fe and subsequently to the nitrogen core. Interestingly, Bi does not significantly participate in hydrogen interactions; no hydrogen atom approaches closer than 2.1 Å to Bi.

The synergistic contribution of multiple elements in providing hydrogen while preventing catalyst saturation likely drives the fast kinetics and high selectivity observed. This results in a significantly higher production rate than single-element catalysts and non-optimized multi-component alloys. A clear example is seen with the hydrogen atom H_3_, which initially bonds with Zn in Step 7, then transfers to Sn and remains associated for three steps. After H_8_ departs, H_3_ bonds to Fe and then attaches to *NH₃ to form the final product NH₄⁺. If, for instance, hydrogen atom H_3_ were not held by Zn and Sn for four steps, it could approach the Fe-H_8_ complex and form hydrogen gas instead of facilitating the step-by-step hydrogenation of the nitrogen molecule. By reserving hydrogen atoms and holding them on standby before feeding them to Fe, the catalyst prevents hydrogen atoms from bonding to form hydrogen gas. This mechanism likely contributes to the nearly 100% FE and seven-fold rate improvement observed in ammonia synthesis with HELMA. This integrated approach minimizes experimental workload and creates a robust feedback loop between theoretical and experimental work. The synthesis protocol developed became essential for obtaining consistent and well-mixed catalysts, enabling precise catalyst engineering for efficient ammonia synthesis.

The formation of a robust hydrogen network significantly enhances reaction kinetics by improving hydrogen transfer efficiency at the interface. By leveraging multiple metal sites such as Fe, Sn, and Ni, the hydrogen atoms are strategically coordinated to optimize their availability. For instance, proton adsorption on other metals, while Fe is occupied surfaces, improves proton distribution and strengthens the H-metal-bond network, which mitigates hydrogen depletion near the active site. This connectivity creates efficient proton transfer channels and reduces the energy barriers for intermediate steps, ensuring a continuous and selective hydrogen supply to the reaction centers. Such optimization of interfacial H-bond networks prevents kinetic bottlenecks, enabling faster hydrogen donation without saturation and facilitating stepwise water molecule formation, which is critical to achieving enhanced kinetics and high selectivity in NO_x_RR and related hydrogenation processes.

**Liquid dynamic environment**

The dynamic environment provided by multi-element electrocatalysts of HELMA significantly enhances complex and multi-step NO_x_RR electrocatalytic reactions by offering tunable adsorption/desorption energies and synergistic interactions among multiple elements. HELMA exhibits unique structural and electronic properties due to its high entropy, lattice distortion (liquid state), and cocktail effects (multi-elements), which result in optimized surface reactivity. The multi-element composition allows continuous electronic structure tuning, enabling precise control over reaction intermediates' binding energy. Additionally, HELMA's nanoscale or sub-nanoscale dimensions maximize active surface sites and promote the exposure of under-coordinated atoms, which are highly active in multi-step reactions. This dynamic surface environment facilitates the stabilization of transient intermediates, lowers reaction barriers, and accelerates reaction kinetics, making HELMA particularly effective for challenging reactions such as the NO_x_RR demonstrated here. Furthermore, its structural stability and anti-aggregation properties ensure durability under high-rate electrochemical conditions, positioning HELMA as an ideal candidate for advancing sustainable energy conversion technologies (Figure S 9).


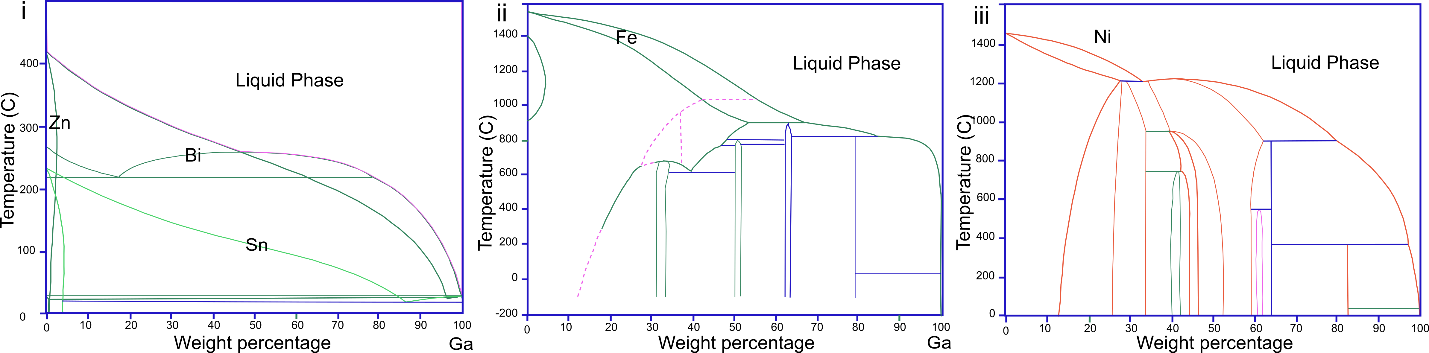


**Figure S1.** Phase diagrams for selected metals (Fe, Bi, Ni, Sn, Zn, and Ga) highlight optimal conditions for forming liquid-phase alloys with Ga. Each alloy composition includes 0.5 wt% of the additional metal blended with Ga. Specifically: **i,** The phase diagrams for Bi, Zn, and Sn with Ga delineate both liquidus and solidus boundaries. They indicate that at low concentrations (0.5 wt%) of these metals, the alloy remains liquid just above room temperature, underscoring the potential to form stable liquid-phase alloys. **ii,** In contrast, the Ga-Fe phase diagram shows that incorporating 0.5 wt% Fe into Ga promotes a solid-phase alloy under similar thermal conditions. **iii,** The Ga-Ni phase diagram reveals that at 0.5 wt% Ni, the alloy can maintain a stable liquid phase at slightly elevated temperatures.


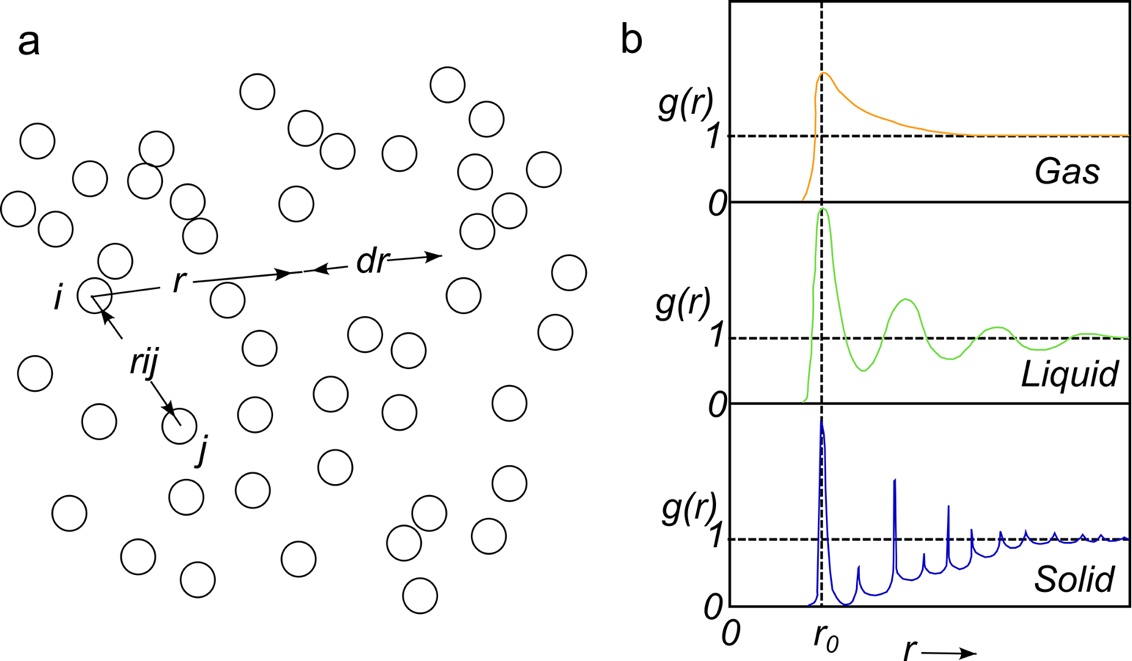


**Figure S2.** Radial Distribution Function (RDF), g(r), the probability of finding two atoms a distance r apart.^7^


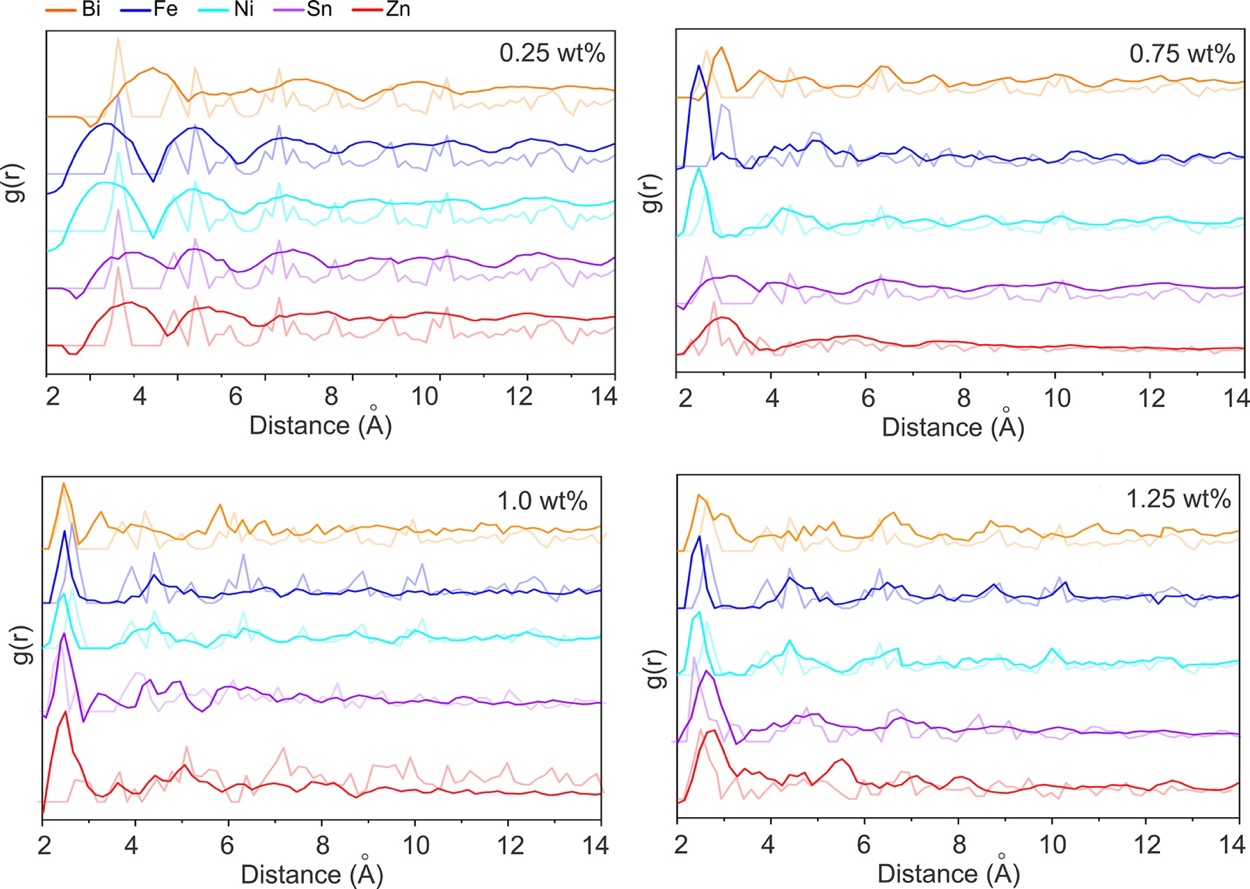


**Figure S3.** MD simulations for finding maximum element concentrations in a liquid alloy. Radial distribution functions g(r) of binary alloys (0.25 to 1.5wt%) at 40 ^°^C (dark-colored) and the inherent structures before transition to liquid (light-colored).


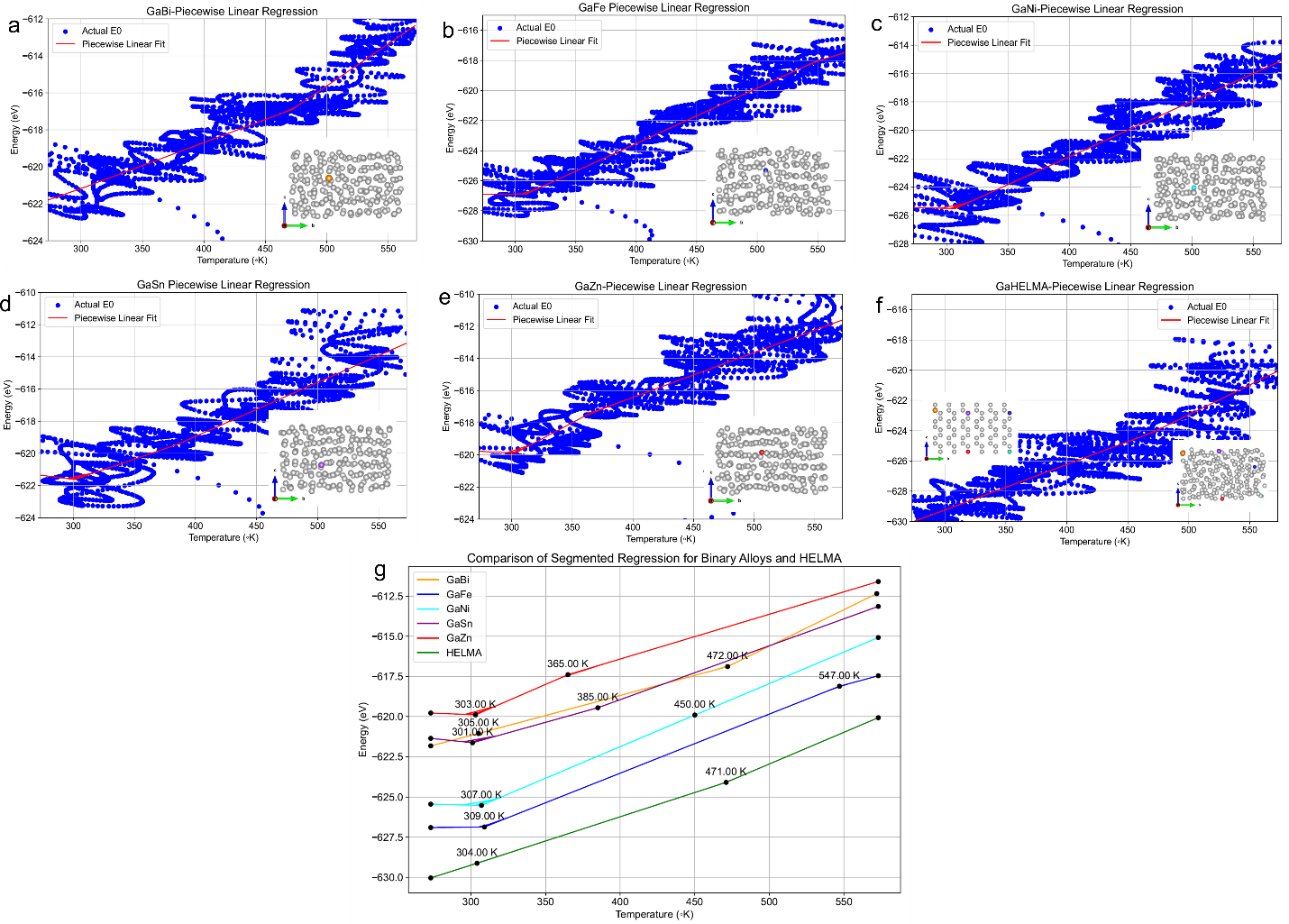


**Figure S4.** **a-f,** MD simulations illustrating the piecewise regression analysis for the five final elements in HELMA as binary alloys with Ga and the HELMA across the temperature range of 273K to 573K. Insets show the structural changes in various binary alloy systems (Fe, Bi, Ni, Sn, Zn) as modeled by MD simulations under increasing temperature from 273K to 573K. The structural arrangement transitions from a more ordered crystalline state to a disordered state, characteristic of melting and increased thermal motion. The directional axes (a, b, c) suggest the orientation of the crystal lattice, and the visual representations highlight how atoms lose their positional coherence as thermal energy disrupts bonding and promotes diffusion. This indicates temperature-induced transformations typical of phase transitions in materials.


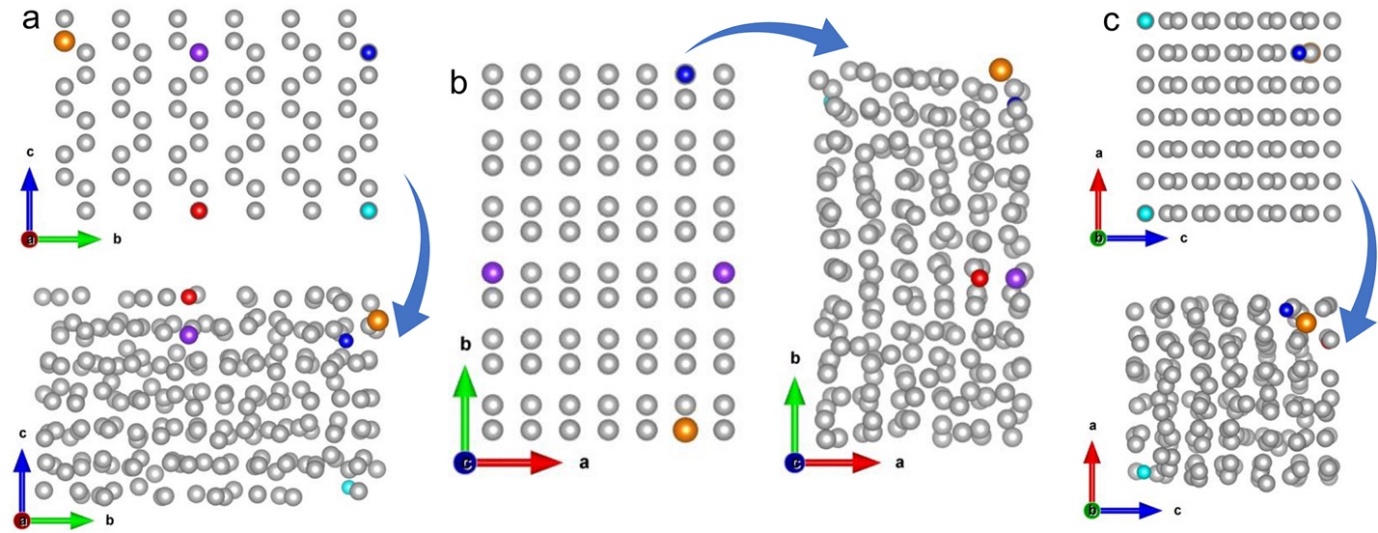


**Figure S5.** **a–c,** Structural changes in HELMA modeled by MD simulations as temperature increases, showing phase transformations to a liquid state occurring around 300 K from different perspectives. The structural arrangement transitions from an ordered crystalline to a disordered state, indicating melting and increased atomic thermal motion. The visualizations along the directional axes (a, b, c) highlight the orientation of the crystal lattice and demonstrate how atoms progressively lose positional coherence as thermal energy disrupts bonding and enhances atomic diffusion. These results provide evidence of temperature-induced transformations characteristic of phase transitions in high-entropy liquid metal alloys.


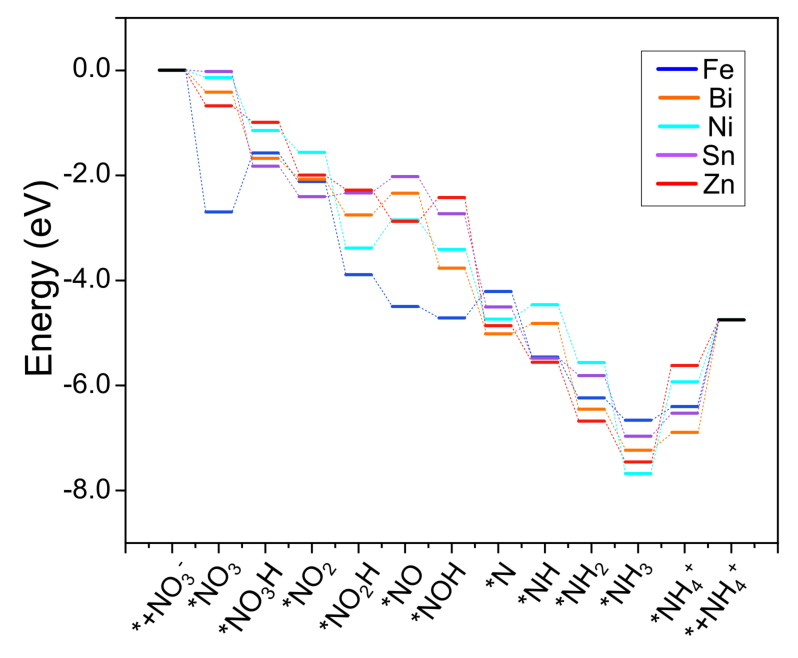


**Figure S6.** DFT calculation results for HELMA, each reaction pathway is color-coded to indicate the active element.


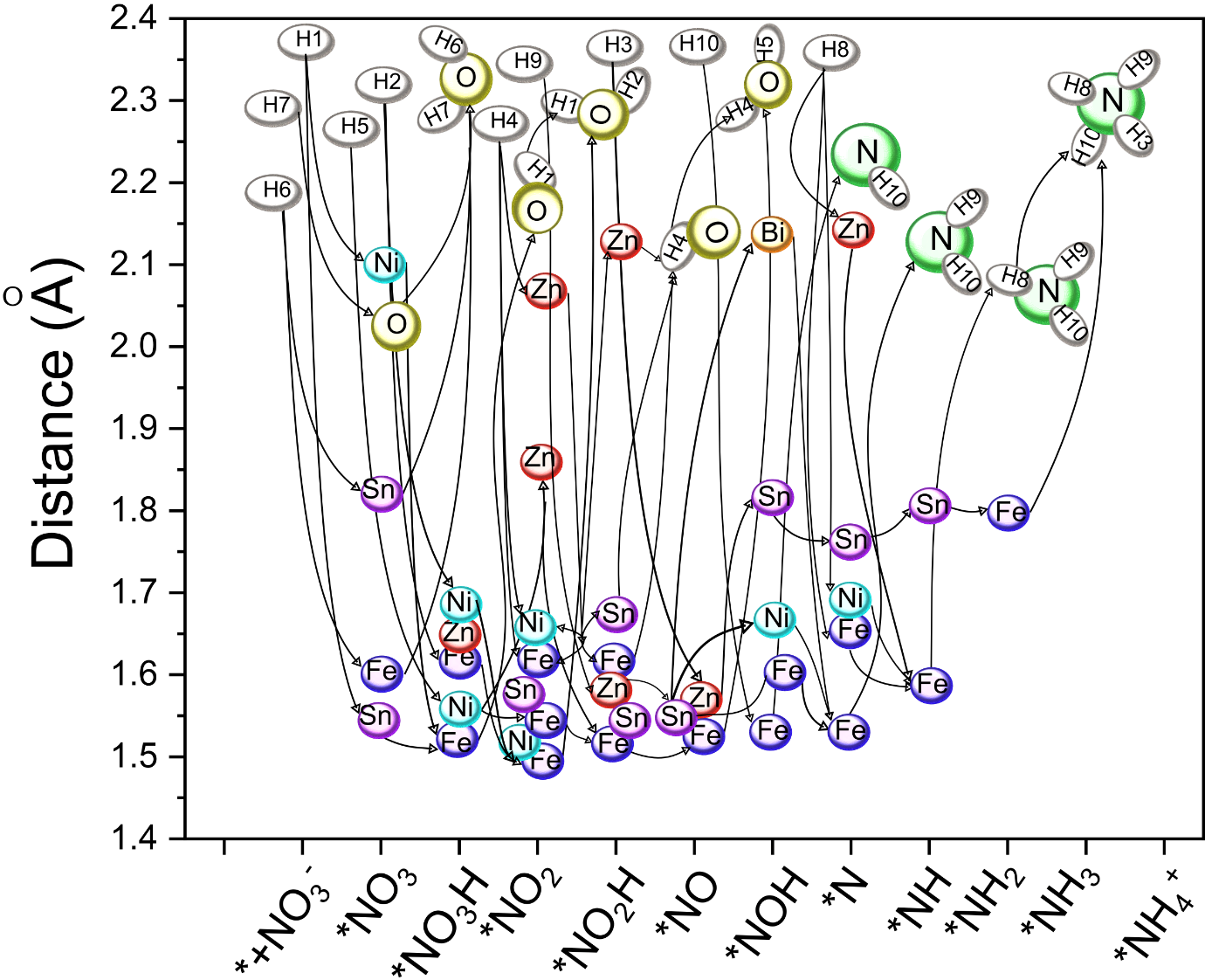


**Figure S7.** Distance between each hydrogen atom (randomly labeled H₁ to H₁₀) and each metal element was calculated at every reaction step. Each column represents the formation of a specific intermediate during the thirteen steps of the NO_x_RR. The y-axis displays the distance between hydrogen atoms and metals, while the x-axis indicates the reaction step. When a hydrogen atom is close to a particular metal, it is depicted as bonded to that metal within the corresponding column. This Figure S7 traces the trajectories of the ten hydrogen atoms, allowing identification of the reactions and products formed through each hydrogen–metal interaction.


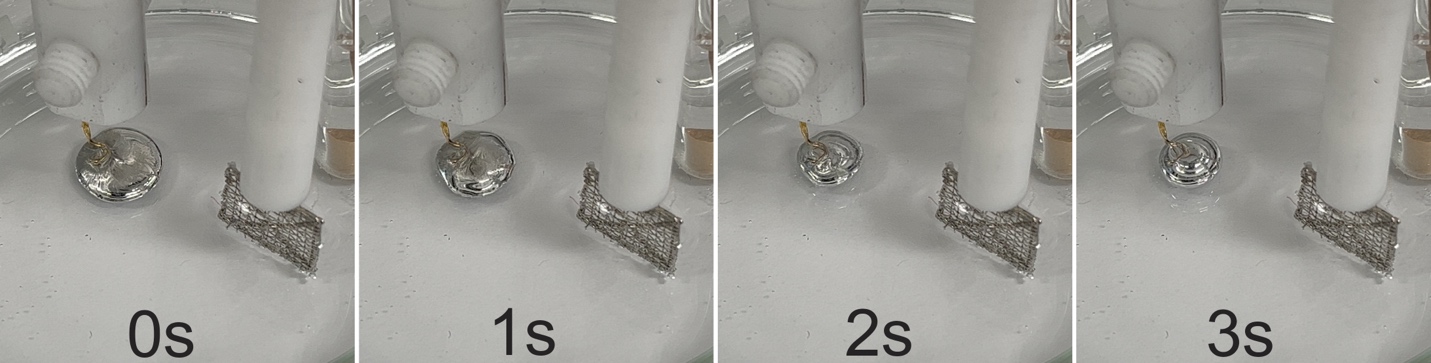


**Figure S8.** Snapshot of the electrocatalysis cell using high-entropy liquid metal alloy for ammonia synthesis within the first few seconds. An oxide skin forms on the catalyst surface upon transferring HELMA to the electrochemical test and adding the electrolyte. Immediately after the current is applied, the oxide skin is removed, allowing the liquid catalyst to regain its high surface tension and form a hemispherical shape. Throughout the experiment, the movement and mobility of the catalyst surface are observable, demonstrating the adaptability and unique features of using a liquid catalyst. This dynamic movement facilitates the continuous mixing of the alloy, ensuring that all elements remain available for the electrocatalytic process.

Additionally, if any elements sink beneath the surface, they are remixed and made accessible, maintaining optimal catalytic performance and enhancing the adaptability of the electrocatalysts. By refining the synthesis protocol to achieve a more homogeneous alloy mixture and utilizing the advantages of a liquid metal alloying (such as simplified synthesis, reproducibility, and consistent physical properties), we established a dynamic environment that enhances NOₓRR catalytic performance. This consistency is vital for integrating experimental and theoretical methods to optimize catalyst design effectively, ensuring that performance variations stem from intrinsic material properties rather than extrinsic factors.


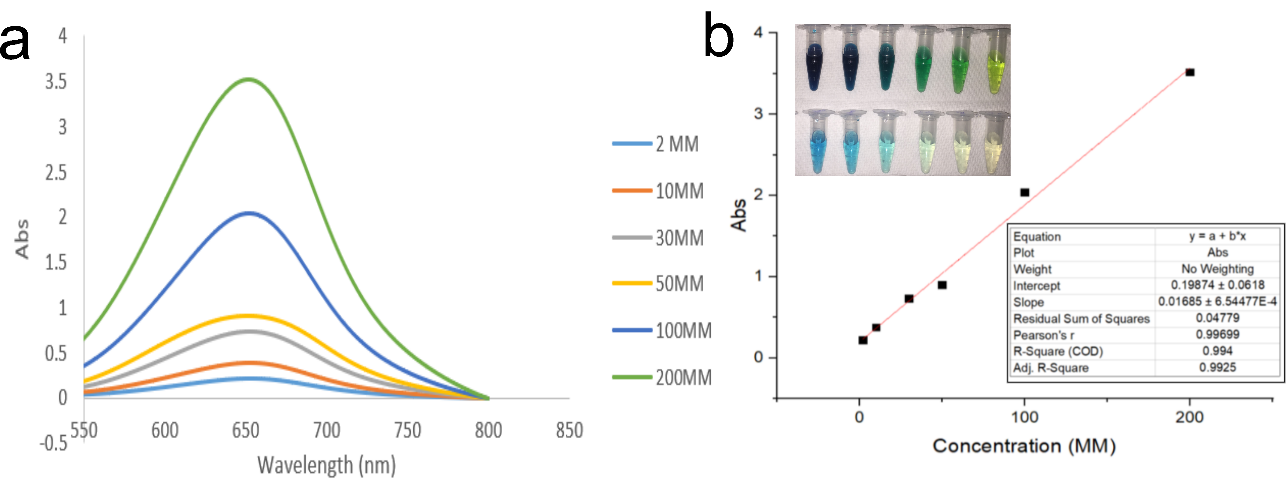


**Figure S9.** Calibration curves were generated using UV-Vis spectroscopy to quantify the concentrations of ammonia and nitrite throughout the study. a, UV-Vis spectroscopy calibrates the Indophenol blue test for precise ammonia detection and quantification. b, The calculated calibration curves from the UV-Vis data.

Concentration=59.3472*Abs-11.7947

**Table S1.**  Alloy compositions are suggested via DoE and synthesized in this research. Partial fraction of each element in the alloy is 0.5wt%.

| Binary | Ga^+^ | Multi-component alloy | Ga^+^ | Multi-component alloy | Ga^+^ |
| --- | --- | --- | --- | --- | --- |
| 1 | Ag | 1 | MoMnRuNiBiW | 16 | ZnInNiBiCuSnW |
| 2 | Bi | 2 | MoRuAgZnNiPdSnW | 17 | MoMnZnInNiFePdSn |
| 3 | Cu | 3 | MoMnRuAgFePd | 18 | MoRuInNiCuFeW |
| 4 | Fe | 4 | MoZn | 19 | MnRuZnNiCuPd |
| 5 | In | 5 | MoMnAgZnNiBiCuFeW | 20 | AgZnInCuFePdSn |
| 6 | Mn | 6 | MoMnRuNiBiSn | 21 | MnRuAgInNiBiCuFePdSnW |
| 7 | Mo | 7 | MoAgInNiBiCuPd | 22 | MoMnRuZnInBiPdW |
| 8 | Ni | 8 | AgZnInCuFePdW | 23 | MoAgInBiFeSnW |
| 9 | Pd | 9 | RuInPdSn | 24 | MnRuZnFeSnW |
| 10 | Ru | 10 | MnAgInNiW | 25 | RuAgZnInNiBiFe |
| 11 | Sn | 11 | MnInBiCuFe | 26 | RuAgBiCuW |
| 12 | W | 12 | AgNiCuFeSn | 27 | MoRuZnBiCuFePdSn |
| 13 | Zn | 13 | MnAgZnBiPdSn | 28 | MoMnRuAgZnInCuSn |
|  |  | 14 | NiBiFePdW | **29** | **HELMA (BiFeNiSnZn)** |
|  |  | 15 | MoMnCuPdSnW |  |  |

**Table S2.** The alloy composition proposed by DoE and its corresponding electrochemical performance measurements (response).

| **Run** | **Alloy composition** | **Element** | | | | | | | | | | | | | **FE** | **Rate** |
| --- | --- | --- | --- | --- | --- | --- | --- | --- | --- | --- | --- | --- | --- | --- | --- | --- |
|  |  | **Mo** | **Mn** | **Ru** | **Ag** | **Zn** | **In** | **Ni** | **Bi** | **Cu** | **Fe** | **Pd** | **Sn** | **W** | **(%)** | **(nmol s^-1^cm^-2^)** |
| 1 | MoMnRuNiBiW | Yes | Yes | Yes | No | No | No | Yes | Yes | No | No | No | No | Yes | 98.75 | 169.66 |
| 2 | MoRuAgZnNiPdSnW | Yes | No | Yes | Yes | Yes | No | Yes | No | No | No | Yes | Yes | Yes | 74.77 | 219.15 |
| 3 | MoMnRuAgFePd | Yes | Yes | Yes | Yes | No | No | No | No | No | Yes | Yes | No | No | 88.24 | 180.86 |
| 4 | MoZn | Yes | No | No | No | Yes | No | No | No | No | No | No | No | No | 65.21 | 132.33 |
| 5 | MoMnAgZnNiBiCuFeW | Yes | Yes | No | Yes | Yes | No | Yes | Yes | Yes | Yes | No | No | Yes | 91.86 | 155.34 |
| 6 | MoMnRuNiBiSn | Yes | Yes | Yes | No | No | No | Yes | Yes | No | No | No | Yes | No | 70.51 | 139.10 |
| 7 | MoAgInNiBiCuPd | Yes | No | No | Yes | No | Yes | Yes | Yes | Yes | No | Yes | No | No | 67.44 | 145.14 |
| 8 | AgZnInCuFePdW | No | No | No | Yes | Yes | Yes | No | No | Yes | Yes | Yes | No | Yes | 91.65 | 113.65 |
| 9 | RuInPdSn | No | No | Yes | No | No | Yes | No | No | No | No | Yes | Yes | No | 76.12 | 156.02 |
| 10 | MnAgInNiW | No | Yes | No | Yes | No | Yes | Yes | No | No | No | No | No | Yes | 95.74 | 124.12 |
| 11 | MnInBiCuFe | No | Yes | No | No | No | Yes | No | Yes | Yes | Yes | No | No | No | 94.52 | 213.12 |
| 12 | AgNiCuFeSn | No | No | No | Yes | No | No | Yes | No | Yes | Yes | No | Yes | No | 84.12 | 113.80 |
| 13 | MnAgZnBiPdSn | No | Yes | No | Yes | Yes | No | No | Yes | No | No | Yes | Yes | No | 99.15 | 158.40 |
| 14 | NiBiFePdW | No | No | No | No | No | No | Yes | Yes | No | Yes | Yes | No | Yes | 98.57 | 140.54 |
| 15 | MoMnCuPdSnW | Yes | Yes | No | No | No | No | No | No | Yes | No | Yes | Yes | Yes | 57.84 | 136.33 |
| 16 | ZnInNiBiCuSnW | No | No | No | No | Yes | Yes | Yes | Yes | Yes | No | No | Yes | Yes | 99.39 | 197.73 |
| 17 | MoMnZnInNiFePdSn | Yes | Yes | No | No | Yes | Yes | Yes | No | No | Yes | Yes | Yes | No | 63.55 | 192.13 |
| 18 | MoRuInNiCuFeW | Yes | No | Yes | No | No | Yes | Yes | No | Yes | Yes | No | No | Yes | 63.82 | 218.81 |
| 19 | MnRuZnNiCuPd | No | Yes | Yes | No | Yes | No | Yes | No | Yes | No | Yes | No | No | 71.58 | 161.38 |
| 20 | AgZnInCuFePdSn | No | No | No | Yes | Yes | Yes | No | No | Yes | Yes | Yes | Yes | No | 99.78 | 125.73 |
| 21 | MnRuAgInNiBiCuFePdSnW | No | Yes | Yes | Yes | No | Yes | Yes | Yes | Yes | Yes | Yes | Yes | Yes | 99.77 | 116.08 |
| 22 | MoMnRuZnInBiPdW | Yes | Yes | Yes | No | Yes | Yes | No | Yes | No | No | Yes | No | Yes | 84.44 | 171.35 |
| 23 | MoAgInBiFeSnW | Yes | No | No | Yes | No | Yes | No | Yes | No | Yes | No | Yes | Yes | 80.58 | 152.78 |
| 24 | MnRuZnFeSnW | No | Yes | Yes | No | Yes | No | No | No | No | Yes | No | Yes | Yes | 52.50 | 106.53 |
| 25 | RuAgZnInNiBiFe | No | No | Yes | Yes | Yes | Yes | Yes | Yes | No | Yes | No | No | No | 97.85 | 120.93 |
| 26 | RuAgBiCuW | No | No | Yes | Yes | No | No | No | Yes | Yes | No | No | No | Yes | 99.19 | 174.30 |
| 27 | MoRuZnBiCuFePdSn | Yes | No | Yes | No | Yes | No | No | Yes | Yes | Yes | Yes | Yes | No | 60.37 | 95.90 |
| 28 | MoMnRuAgZnInCuSn | Yes | Yes | Yes | Yes | Yes | Yes | No | No | Yes | No | No | Yes | No | 69.31 | 125.02 |
| 29 | GaAg |  |  |  |  |  |  |  |  |  |  |  |  |  | 78.79 | 64.60 |
| 30 | GaBi |  |  |  |  |  |  |  |  |  |  |  |  |  | 99.92 | 115.96 |
| 31 | GaCu |  |  |  |  |  |  |  |  |  |  |  |  |  | 85.14 | 157.05 |
| 32 | GaFe |  |  |  |  |  |  |  |  |  |  |  |  |  | 35.22 | 131.01 |
| 33 | GaGa |  |  |  |  |  |  |  |  |  |  |  |  |  | 73.94 | 74.89 |
| 34 | GaIn |  |  |  |  |  |  |  |  |  |  |  |  |  | 72.99 | 65.83 |
| 35 | GaMn |  |  |  |  |  |  |  |  |  |  |  |  |  | 87.51 | 76.23 |
| 36 | GaMo |  |  |  |  |  |  |  |  |  |  |  |  |  | 43.69 | 78.81 |
| 37 | GaNi |  |  |  |  |  |  |  |  |  |  |  |  |  | 97.59 | 108.84 |
| 38 | GaPd |  |  |  |  |  |  |  |  |  |  |  |  |  | 91.81 | 65.20 |
| 39 | GaRu |  |  |  |  |  |  |  |  |  |  |  |  |  | 25.22 | 59.44 |
| 40 | GaSn |  |  |  |  |  |  |  |  |  |  |  |  |  | 99.39 | 102.16 |
| 41 | GaW |  |  |  |  |  |  |  |  |  |  |  |  |  | 58.37 | 59.82 |
| 42 | GaZn |  |  |  |  |  |  |  |  |  |  |  |  |  | 97.70 | 139.29 |
| 43 | HELMA | No | No | No | No | Yes | No | Yes | Yes | No | Yes | No | Yes |  | 99.08 | 315.97 |

**Table S3.**  Energy barrier of NO_x_RR for binary alloys.

| Energy barrier (eV) | Ga | GaBi | GaFe | GaNi | GaSn | GaZn | GaCu | GaMo | GaMn | GaRu | GaAg | GaIn | GaPd | GaW |
| --- | --- | --- | --- | --- | --- | --- | --- | --- | --- | --- | --- | --- | --- | --- |
| Sum of endothermic steps | 5.37 | 6.39 | 5.34 | 5.55 | 2.95 | 8.39 | 2.89 | 7.54 | 7.8 | 5.37 | 5.32 | 5.33 | 5.23 | 7.36 |
| Activation energy | 5.06 | 3.33 | 1.92 | 2.2 | 1.82 | 4.27 | 1.94 | 2.86 | 3.65 | 2.16 | 2.01 | 2.09 | 2.26 | 3.21 |
| H* | -1.54 | -0.37 | 0.51 | 0.27 | -0.1 | -0.14 | 0.56 | -1.55 | -0.84 | -0.32 | 0.04 | 0.36 | 0.74 | -1.44 |

**Table S4.**  Interaction energies between intermediates and each element at every reaction step

| Step | Intermediate | Approaching head | GaBi (eV) | GaFe (eV) | GaNi (eV) | GaSn (eV) | GaZn (eV) |
| --- | --- | --- | --- | --- | --- | --- | --- |
| 1 | *+NO_3_^-^ | Zero state | -111.70 | -116.89 | -114.46 | -112.76 | -109.57 |
| 2 | NO_3_* | NO3+* | -111.35 | -115.13 | -112.26 | -112.31 | -109.62 |
| 3 | * NO_3_H | HE | -111.87 | -114.89 | -113.85 | -73.58 | -108.29 |
|  |  | HGa | -111.57 | -81.21 | -112.69 | -111.80 | -109.27 |
|  |  | NE | -111.84 | -117.61 | -113.75 | -75.00 | -108.87 |
|  |  | NGa | -113.48 | -116.28 | -113.81 | -111.48 | -111.13 |
|  |  | OGa+OE | -113.66 | -117.50 | -115.10 | -112.03 | -109.65 |
| 4 | *NO_2_ | NE | -102.91 | -72.43 | -104.14 | -103.01 | -99.59 |
|  |  | NGa | -103.41 | -105.63 | -104.61 | -102.80 | -100.05 |
|  |  | OGa+OE | -102.49 | -70.96 | -104.80 | -103.07 | -100.51 |
|  |  | OGa+OE+NGa | -102.23 | -21.96 | -105.00 | -102.85 | -100.78 |
|  |  | OGa+OGa+NE | -102.65 | -108.07 | -105.34 | -102.42 | -100.52 |
| 5 | *NO_2_H | HE | -105.95 | -109.10 | -107.75 | -105.49 | -103.15 |
|  |  | HGa | -106.24 | -108.83 | -107.17 | -105.93 | -103.38 |
|  |  | NE | -106.34 | -75.02 | -107.61 | -106.19 | -102.80 |
|  |  | NGa | -106.03 | -111.01 | -108.20 | -105.48 | -103.23 |
|  |  | OGa+OE | -105.75 | -76.34 | -107.61 | -106.06 | -102.50 |
| 6 | *NO | NE | -95.68 | -65.25 | -99.02 | -96.27 | -93.41 |
|  |  | NGa | -95.53 | -99.44 | -97.73 | -94.95 | -93.08 |
|  |  | OE | -95.88 | -64.76 | -96.66 | -95.38 | -92.51 |
|  |  | OE+NGa | -96.25 | -37.30 | -98.95 | -96.40 | -93.67 |
|  |  | OGa | -95.37 | -98.93 | -97.38 | -95.70 | -92.60 |
|  |  | OGa+NE | -96.12 | -56.54 | -98.31 | -96.49 | -92.87 |
| 7 | *NOH | HGa+OE | -99.57 | -69.32 | -101.24 | -99.53 | -97.50 |
|  |  | NE | -99.51 | -104.89 | -101.75 | -99.71 | -97.05 |
|  |  | NGa | -100.74 | -103.56 | -102.05 | -99.45 | -97.07 |
|  |  | OGa+HE | -100.09 | -104.43 | -102.27 | -99.09 | -97.75 |
|  |  | OGa+HE+NGa | -100.31 | -104.84 | -103.36 | -100.76 | -97.42 |
|  |  | OGa+HGa+NE | -99.31 | -104.67 | -101.21 | -99.15 | -97.08 |
| 8 | *N | NE | -90.60 | -94.35 | -92.32 | -91.19 | -80.21 |
|  |  | NGa | -90.48 | -94.23 | -54.06 | -89.89 | -49.50 |
| 9 | *NH | HE | -94.59 | -12.99 | -97.17 | -94.84 | -59.81 |
|  |  | HE+NGa | -94.49 | -13.80 | -96.63 | -94.81 | -91.35 |
|  |  | HGa | -95.22 | -63.56 | -97.36 | -93.47 | -92.05 |
|  |  | HGa+NE | -95.29 | -63.16 | -95.47 | -93.60 | -91.98 |
|  |  | NE | -94.99 | -62.55 | -97.11 | -93.73 | -92.68 |
|  |  | NGa | -95.18 | -62.72 | -96.34 | -94.00 | -92.31 |
| 10 | *NH_2_ | HGa+HE | -100.23 | -67.08 | -100.42 | -99.04 | -95.41 |
|  |  | HGa+HE+NGa | -99.97 | -67.62 | -101.12 | -98.91 | -95.25 |
|  |  | HGa+HGa+NE | -101.20 | -67.14 | -101.24 | -99.16 | -96.74 |
|  |  | NE | -98.85 | -92.90 | -100.68 | -98.60 | -96.30 |
|  |  | NGa | -98.91 | -67.50 | -101.76 | -98.43 | -95.05 |
| 11 | *NH_3_ | EH-GaH | -102.92 | -71.70 | -103.98 | -102.94 | -100.50 |
|  |  | NE | -102.40 | -106.62 | -104.33 | -102.76 | -100.80 |
|  |  | NGa | -102.43 | -105.71 | -104.53 | -103.61 | -100.25 |
| 12 | *NH_4_^+^ | NE | -111.02 | -113.66 | -112.26 | -110.57 | -107.68 |
|  |  | NGa | -108.44 | -109.39 | -107.32 | -106.49 | -103.20 |
| 13 | *+NH_4_^+^ |  | -107.69 | -112.88 | -110.45 | -108.75 | -105.56 |
| probability of NE being the most favorable approaching head | | | 0.5 | 0.7 | 0.4 | 0.5 | 0.5 |

**Note: Table S4.** shows the interaction energies between intermediates and each element (O = oxygen, N = nitrogen, H = hydrogen, Ga = gallium, E = catalyst element) at every reaction step. Energies were calculated for all possible orientations of the catalyst and the approaching head, with each orientation's energy presented to evaluate and identify the most favorable approach. The "Approaching Head" column specifies the orientation of elements and intermediates used in each calculation. The lowest interaction energy is highlighted as the most probable reaction pathway for each element and step.

**Movie 1.** Gif animation of the calculated NO_x_RR pathway, modeled as slabs containing 32 atoms, including Ga, nitrate, one atom from each of the five metals (Fe, Sn, Zn, Ni, Bi), and ten hydrogen atoms.

**Calculation of entropy**

High entropy catalysts offer significant advantages over traditional catalysts primarily due to their profound configurational disorder, which arises from multiple elements occupying the same crystallographic site in near-equiatomic ratios. This extreme disorder creates a vast number of possible microstates, enhancing the configurational entropy, which contributes to the stabilization of unique phases not accessible in conventional systems. The structural and chemical heterogeneity of high entropy catalysts leads to a broader range of active sites with varied coordination environments, promoting catalytic activity and selectivity. Additionally, diverse elements enable the synergistic "cocktail effect," where the combination of constituents can enhance functional properties, such as thermal stability, resistance to poisoning, and tunable electronic properties, surpassing the performance of conventional single-element catalysts. These features make high entropy catalysts highly versatile and robust for applications in emerging technologies, including electrocatalysis presented here.

The hard-sphere model is a valuable tool for calculating the various thermodynamic properties of liquid metals and their alloys. In this study, we employed this model to calculate the entropy of liquid metals. We assumed that the density of the mixture remains unaffected at room temperature and that the number of microstates remains constant for all alloys, disregarding any influence from structural factors. The Calculation was described in detail in:^8^

$S=S_{0}-S_{1}$ S Eq 13

where S_0_ is the entropy of an ideal gas with the parameters of a liquid metal:

$S_{0}=\frac{5}{2}R+\frac{3}{2}Rln\left[ \frac{mkT\nu^{\frac{2}{3}}}{2\pi h^{2}N^{\frac{2}{3}}} \right]$ S Eq 14

where R is the gas constant, m is the mass of a single microstructure (atom), and k is the Boltzmann constant, ν=µ/ρ (where µ is the molar weight of the microstructure and ρ is the density at room temperature (given temperature)), h is the Planck constant, and N is the Avogadro number, where S_1_ is the configuration entropy (the ratio of the volume of bodies in a space to the volume of the space):

$S_{1}=R\left[ \frac{4n-3n^{2}}{\left( 1-n \right)^{2}}-2n \right]$ S Eq 15

Where n is the density of atom packing (the mass of the atoms in the unit cell).

$\beta_{s}=\frac{1}{{\rho a}^{2}}$ S Eq 16

Where 𝛽_s_ is adiabatic compressibility, ρ is melt density, a is ultrasonic velocity.

$\gamma=1+\frac{\alpha^{2}}{\rho\beta_{s}C_{P}}$ S Eq 17

γ is the Poisson ratio, where α is the thermal expansion coefficient, C_P_ is specific heat, and

$\beta_{T}=\gamma\beta_{s}$ S Eq 18

𝛽_T_ is isothermal compressibility. The structural factor in the statistical theory for the long-wave limit is:

$S_{(0)}=n_{0}k\beta_{T}$ S Eq 19

Where n_0_ is the number of particles in the unit volume. In the rigid-sphere model, the structural factor is associated with the density of atom packing as:

$S_{(0)}={(1-n)}^{4}{(1+2n)}^{-2}$ S Eq 20

It therefore follows that:

$n=1+{(S_{\left( 0 \right)})}^{1/2}-{[3{{(S}_{\left( 0 \right)})}^{1/2}+S_{\left( 0 \right)}]}^{1/2}$ S Eq 21

Based on evidence from the structure factor, most liquid metals behave like hard spheres.^9^

**References**

1. Ding, J., Ma, E., Asta, M. & Ritchie, R. O. Second-nearest-neighbor correlations from connection of atomic packing motifs in metallic glasses and liquids. *Sci Rep* **5**, 17429 (2015). <https://doi.org/10.1038/srep17429>.

2. Kresse, G. & Joubert, D. From ultrasoft pseudopotentials to the projector augmented-wave method. *Phys Rev B* **59**, 1758 (1999). <https://doi.org/10.1103/PhysRevB.59.1758>.

3. Kresse, G. & Hafner, J. Ab initio molecular-dynamics simulation of the liquid-metal–amorphous-semiconductor transition in germanium. *Phys Rev B* **49**, 14251 (1994). <https://doi.org/10.1103/PhysRevB.49.14251>.

4. Kresse, G. & Furthmüller, J. Efficient iterative schemes for ab initio total-energy calculations using a plane-wave basis set. *Phys Rev B* **54**, 11169 (1996). <https://doi.org/10.1103/PhysRevB.54.11169>.

5. Perdew, J. P., Burke, K. & Ernzerhof, M. Generalized gradient approximation made simple. *Phys Rev Lett* **77**, 3865 (1996). <https://doi.org/10.1103/PhysRevLett.77.3865>.

6. Peterson, A. A., Abild-Pedersen, F., Studt, F., Rossmeisl, J. & Nørskov, J. K. How copper catalyzes the electroreduction of carbon dioxide into hydrocarbon fuels. *Energy Environ Sci* **3**, 1311–1315 (2010). <https://doi.org/10.1039/C0EE00071J>.

7. Tester, J. W. & Modell, M. Thermodynamics and its Applications. *(No Title)* (1997).

8. Tekuchev, V. V, Kalinkin, D. P. & Ivanova, I. V. Calculating the Entropy of Solid and Liquid Metals, Based on Acoustic Data. *Russian Journal of Physical Chemistry A* **92**, 819–822 (2018). <https://doi.org/10.1134/S003602441805031X>.

9. Jakse, N. & Pasturel, A. Excess entropy scaling law for diffusivity in liquid metals. *Sci Rep* **6**, 20689 (2016). <https://doi.org/10.1038/srep20689>.
