# Supplementary figures and images for: Configuring a Liquid State High‐Entropy Metal Alloy Electrocatalyst
Source: Small. 2025 Jun 17;21(32):2504087. doi: 10.1002/smll.202504087 (PMC12366271; doi:10.1002/smll.202504087)

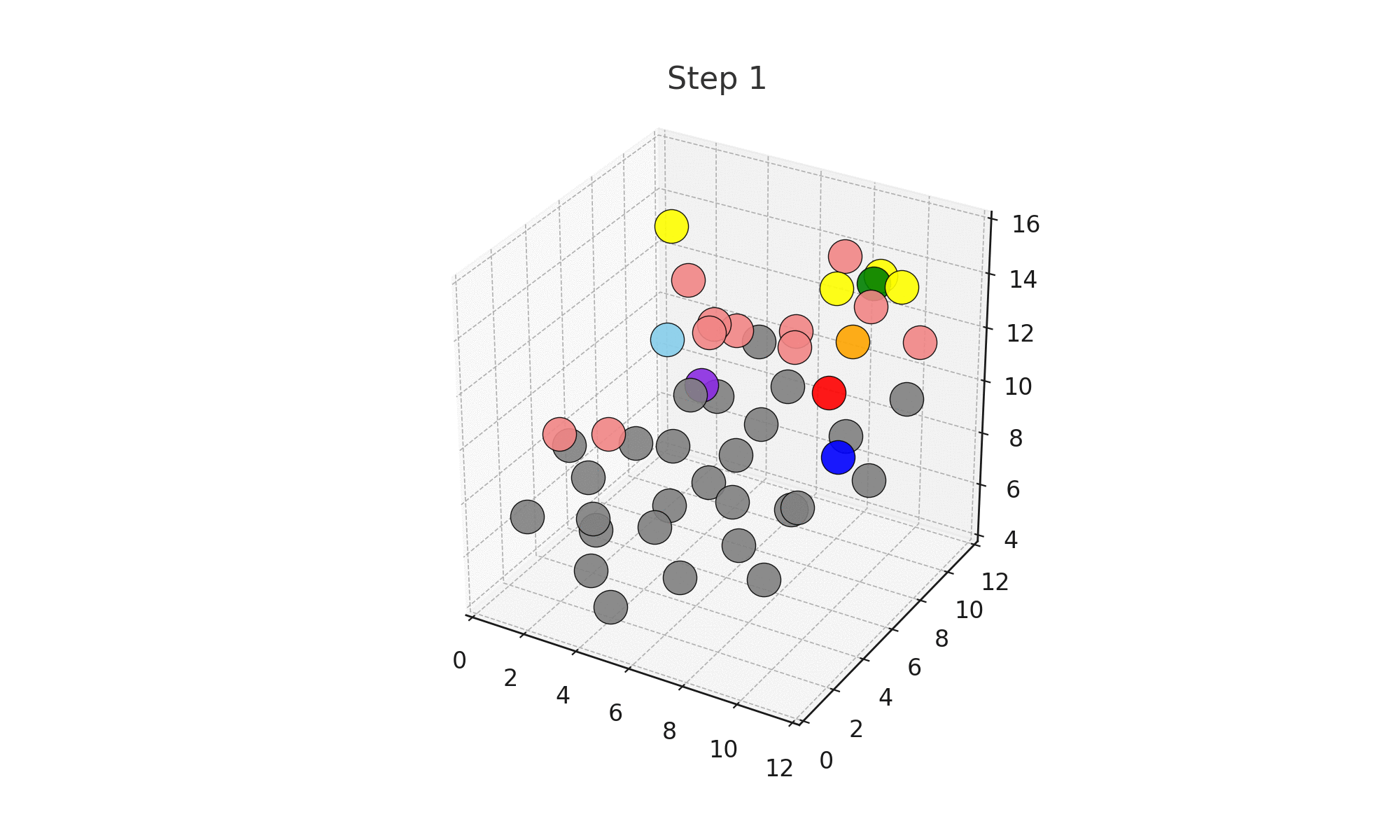

Supplement: Supplementary file 2 — Supplemental Movie 1 [file SMLL-21-2504087-s001.gif]
